# Autophagy dysfunction in iPSCs-derived neurons and midbrain organoids carrying a *SNCA* triplication

**DOI:** 10.1038/s41531-026-01330-x

**Published:** 2026-03-31

**Authors:** Catarina Serra-Almeida, Javier Jarazo, Gemma Gomez-Giro, Isabel Rosety, Alise Zagare, Daniele Ferrante, Cláudia Saraiva, Daniela Frangenberg, Jennifer Modamio-Chamarro, Elisa Zuccoli, Ana Clara Cristóvão, Liliana Bernardino, Jens Christian Schwamborn

**Affiliations:** 1 RISE-Health, Department of Medical Sciences, Faculty of Health Sciences, University of Beira Interior, Av. Infante D. Henrique, 6200-506 Covilhã, Portugal; 2 Luxembourg Center for Systems Biomedicine, University of Luxembourg, Esch-sur-Alzette, Luxembourg; 3 OrganoTherapeutics SARL, Esch-sur-Alzette, Luxembourg; 4 NeuroSoV, UBImedical, University of Beira Interior, Covilhã, Portugal

**Keywords:** Cell biology, Neurology, Neuroscience, Stem cells

## Abstract

Parkinson’s disease (PD), characterized by α-Synuclein aggregation and dopaminergic neuronal loss, has no current cure. Autophagy is critical for α-Synuclein clearance, yet its real-time dynamics remain challenging to assess in human-relevant systems. Here, we used live-cell imaging to assess autophagy within human neuronal cultures and midbrain organoids (hMOs) derived from induced pluripotent stem cells (iPSCs) of PD patients carrying a triplication of the α-Synuclein gene (3xSNCA). Using the LC3-Rosella dual-fluorescent reporter, we quantified autolysosomes dynamics in real time. In 3xSNCA neuronal cultures, we detected early autophagy defects. In 3xSNCA hMOs, reduced autolysosome area, increased total and phosphorylated α-Synuclein (pS129), and decreased electrophysiological activity were observed at 50 days of differentiation (DoD). By 70 DoD, autophagy impairment became more pronounced, overlapping with dopaminergic neuron dysfunction. These findings support the use of human iPSCs-derived models to study autophagy dysfunction in PD and demonstrate a temporal correlation between impaired autophagy, α-Synuclein pathology and neuronal degeneration.

## Introduction

Affecting nearly 10 million people globally, Parkinson´s disease (PD) is a neurodegenerative condition that progresses over time. It belongs to the synucleinopathies, a group of disorders marked by abnormal accumulation of α-Synuclein in several brain areas. Encoded by the *SNCA* gene, this small protein is mostly found in the presynaptic terminals of neurons, where it plays a role in neurotransmitter release^1,2^.

*SNCA* point mutations, such as A53T, A30P, and E46K, as well as gene duplications and triplications are linked with familial PD forms^3–8^. In particular, *SNCA* triplication (3xSNCA) is correlated with elevated α-Synuclein levels and early-onset forms of PD^7,9,10^. Even in idiopathic PD in which genetic mutations have not been identified, α-Synuclein accumulation is observed^11^. Since α-Synuclein aggregation plays a major part in both genetic and sporadic forms of PD, it is imperative to identify the cellular mechanisms that control its expression, aggregation, and clearance. Such insights are critical for advancing our understanding of disease pathogenesis and for identifying novel therapeutic targets in PD.

Autophagy is a key cellular process in the regulation of α-Synuclein levels. It includes a number of pathways such as chaperone-mediated autophagy (CMA) and macroautophagy, both of which are implicated in PD pathogenesis^12,13^. CMA depends on specific proteins like heat shock cognate 70 (HSC70) and lysosomal-associated membrane protein 2 A (Lamp2A) to degrade cytosolic proteins containing the KFREQ motif including wild-type α-Synuclein^14^. Yet, mutant α-Synuclein impairs this pathway which leads to macroautophagy as a compensatory mechanism^15,16^. Macroautophagy involves the delivery of cellular waste, including misfolded or aggregated proteins like α-Synuclein, to lysosomes, where acidic enzymes are responsible for their breakdown. This process depends on the fusion of autophagosomes (double-membraned vesicles) with lysosomes to form autolysosomes^12^.

The accretion of α-Synuclein aggregates together with other pathological mechanisms, such as lysosomal dysfunction, contribute to the degeneration of dopaminergic neurons in *substantia nigra pars compacta*, which are particularly vulnerable^17^. This interaction is interesting since it seems to show a two-way link. The accumulation of α-Synuclein can hinder autophagy, hence facilitating more its aggregation.

Evidence from post-mortem PD brains points to defective autophagy, showing increased autophagosomes, abnormal lysosomes, and high LC3II levels. Lewy bodies positive for LC3 have also been detected in the *substantia nigra*^18^. Furthermore, reduced levels of HSC70 and Lamp2A have also been observed^19^. Similar findings were observed in animal models^20^. Studies have shown that lysosomal defects can impair macroautophagy, leading to the buildup of α-Synuclein^21,22^. These results confirm that autophagy is disrupted in PD, across models and species. Still, little is known whether these impairments are a cause or a consequence of α-Synuclein accumulation.

Therefore, it is important to unravel this relationship by identifying human-relevant models that allow real-time tracking of autophagic dynamics. Advances in induced pluripotent stem cells (iPSCs) technology led to the development of patient-specific neuronal cell culture and organoid models that replicate key features of PD^23^. Although iPSCs-derived neuronal cultures provide a practical and accessible model for PD modelling^24^, their two-dimensional (2D) architecture lacks cell-cell and cell-extracellular matrix (ECM) interactions and midbrain complexity^25,26^. In contrast, three-dimensional (3D) organoids are a more advanced system that can self-organise into structures that resemble the architecture and function of human organs^27^. Specifically, human midbrain organoids (hMOs) can generate a large number of dopaminergic neurons and glial cells, and release dopamine^28,29^. Several studies have used iPSCs-based cell models to explore *SNCA*-related mechanisms in PD^30^. Our research group, alongside others, has shown that iPSCs-derived hMOs carrying a 3xSNCA show dopaminergic neuron loss and α-Synuclein aggregation^31–34^. This evidence indicates their relevance as PD models.

However, even with growing evidence of autophagy disruption in PD, most studies rely on static endpoints analysis, which do not capture the progression and the timing of the impairments. Hence, the temporal dynamics of autophagy in human 3xSNCA models remain poorly understood, representing a major gap for the field. To tackle this limitation, we implemented the LC3-Rosella system, a dual-fluorescent construct enabling the visualization of autolysosomes in live cells. By applying this tool to both neuronal cultures and hMOs derived from iPSCs, we aimed to investigate how autophagy changes throughout disease progression in these models.

In PD neuronal cultures, autophagy exhibits dynamic modulation over the course of time. Autophagic activity was found decreased in the beginning of neuronal differentiation, followed by transient increase and subsequent decline. In contrast, PD hMOs exhibited a progressive and sustained reduction in autophagy, particularly at 70 days of differentiation (hereafter referred to as DoD). PD hMOs also exhibited increased total and phosphorylated α-Synuclein (pS129) in addition to its aggregation at these later timepoints (50 and 70 DoD), followed by dopaminergic neuron dysfunction. This temporal correlation suggests that autophagy failure may precedes and may possibly contributes to pathological α-Synuclein inclusions. Altogether, these findings reinforce the link between autophagy impairment and α-Synuclein pathology.

## Results

### PD neuronal cultures show early and progressive autophagy impairment

This study is based on several human iPSCs lines, as detailed in Supplementary Table 1. The PD patient line, carrying a 3xSNCA, was edited to incorporate the autophagy sensor (PD-auto), created by integrating the LC3-Rosella sensor^35^. For comparison, we included two sex- and age-matched healthy control lines: a standard control (Control) and a modified control (Control-auto) that also incorporates the autophagy sensor. All experimental iPSC lines in this study are classified as either Control or PD lines. The group names “Control-auto” and “PD-auto,” as listed in Supplementary Table 1, refer to Control or PD lines that have been engineered to express the LC3-Rosella autophagy reporter. We previously showed that all iPSCs lines exhibited embryonic stem cell-like morphology and expressed pluripotency markers (SOX2, OCT4, NANOG)^33^.

iPSCs were differentiated into neuroepithelial stem cells (NESCs) with a hindbrain/midbrain identity, as we previously described^36^. To confirm neural stem cell identity, NESCs were characterized by the expression of neural progenitor markers, including SOX1, SOX2, Nestin and PAX6, which indicates dorsal identity (Supplementary Fig. 1; see Supplementary Table 2 for the list of antibodies). Neuronal cultures were then generated from these NESCs and maintained for up to 11 DoD. The schematic representation of neuronal cultures generation is in Supplementary Fig. 2.

**Fig. 1:**
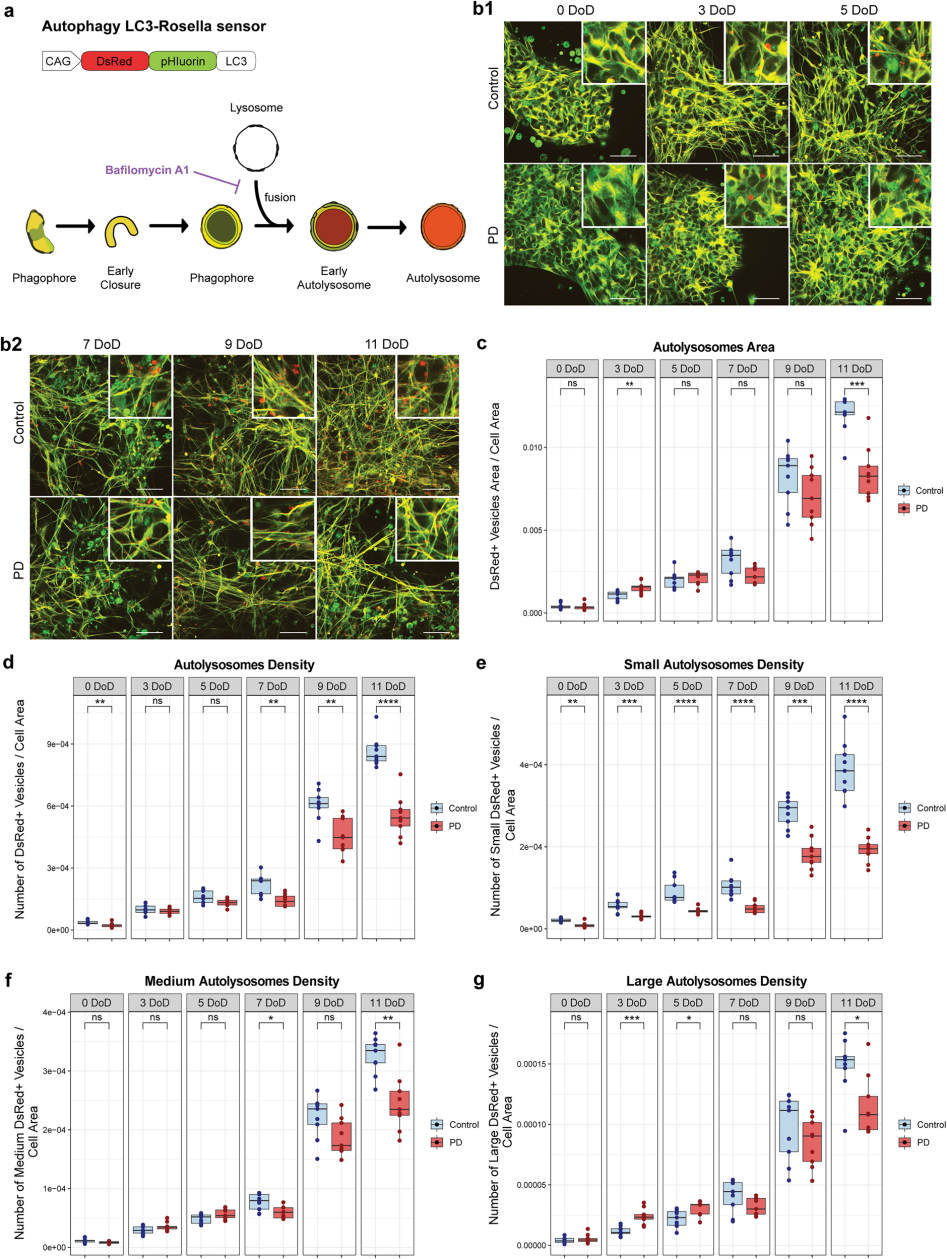
Autolysosome size and density in PD neurons over 11 DoD. **a** Structure of LC3-Rosella sensor, using the pH-sensitive pHluorin and DsRed proteins. It allows us to distinguish autolysosomes (pHluorin-DsRed+ structures) in our cellular model. **b1**, **b2** Representative live-cell microscopy images of neuronal cultures expressing the LC3-rosella autophagy reporter. Images show control and PD neurons at all stages of differentiation, with their respective zoomed region of interest. Scale bar: 20 μm. (**c**-**g**) Quantitative analysis of autophagy in neuronal cultures using the LC3-rosella reporter, at 0, 3, 5, 7, 9 and 11 DoD. PD neurons showed dynamic autophagy impairment over time compared to control. **c** Total autolysosome area per cell area. **d** Number of autolysosomes per cell area. Number of small (**e**), medium (**f**), and large (**g**) autolysosomes per cell area. Data result from three independent experiments each analysed in triplicate. Values are normalized to the average of controls across all time points, allowing a comparison over time. Statistical analysis was performed using Wilcoxon *T*-test; *p < 0.05, **p < 0.01, ***p < 0.001, and ****p < 0.0001, with “ns” indicating non-significant results. Image analysis was performed using a custom MATLAB script for automated quantification of the different parameters of the autolysosome analysis.

To investigate early autophagy dynamics, we first analysed iPSCs-derived 2D neuronal cultures using the LC3-Rosella autophagy sensor, which incorporates the DsRed and pHluorin fluorescent proteins. In acidic environments, like the autolysosome lumen, pHluorin is quenched while DsRed remains fluorescent, indicating fusion with lysosomes^35^. This allowed us to identify autolysosomes that appeared as red vesicles (Fig. 1a). Representative images in Fig. 1b1, b2 show the progressive reduction in red-only vesicles in PD neurons, indicative of impaired autophagy over time. We assessed the area and density of autolysosomes, which were further classified as small, medium, and large, based on their size distribution. The size of these structures, described in the methods section, can be used as an indicator of their functional state. Small autolysosomes are usually more efficient in degrading materials, while larger ones suggest decreased degradation capacity^37,38^.

At 0 DoD, total autolysosome area was similar, but PD cultures had fewer small autolysosomes (Fig. 1c-e), suggesting early degradation defects. No differences were observed in the density of medium and large autolysosomes at this stage (Fig. 1f, g). At 3 DoD, PD cultures exhibited a higher autolysosome area compared to controls, with no difference in the overall autolysosome density (Fig. 1c, d). At the same time, PD neurons had more large autolysosomes (Fig. 1g) but fewer small ones (Fig. 1e), that might indicate an accumulation of autolysosomes that failed to degrade their contents properly. At 5 DoD, no differences were observed in the total autolysosome area and the density of medium autolysosomes in PD neurons (Fig. 1c, f). However, PD neurons showed a persistently lower density of small autolysosomes and a higher density of large autolysosomes (Fig. 1e, g). By 7 DoD, while the total autolysosome area showed no significant difference between PD and control cultures, we observed a lower autolysosome density across all size categories in PD neurons (Fig. 1c-g), indicating that autophagic activity worsened over time. At late time points of differentiation (9 and 11 DoD), PD cultures showed reduced autolysosome area and density across all sizes in comparison to controls (Fig. 1c-g).

These data indicate progressively impaired autophagy in PD neurons, which begins early and worsens over time. These findings highlight a strong correlation between autophagy impairment and PD progression, and suggest that targeting autophagy, particularly at early stages, may have therapeutic potential.

### PD hMOs recapitulate autophagy dysfunction observed in PD neuronal cultures

Having established the presence of early autophagic impairment in neuronal cultures, we next sought to assess whether similar alterations occurred in hMOs, a more physiologically relevant context. These 3D structures mimic several aspects of human midbrain and cellular organisation, making them suitable for studying long-term autophagic dynamics^27–29,33^. The cellular composition of hMOs utilized in this study has been previously characterized by our group^29,33,39^. Accordingly, they contain dopaminergic neurons (TH + , DAT + , NURR1 + ), neural progenitors (SOX2 + ), mature neurons (MAP2 + ), astrocytes (GFAP + , S100β + ), and oligodendrocyte lineage cells (MBP + , CNPase + ), as shown in our previous studies.

**Fig. 2:**
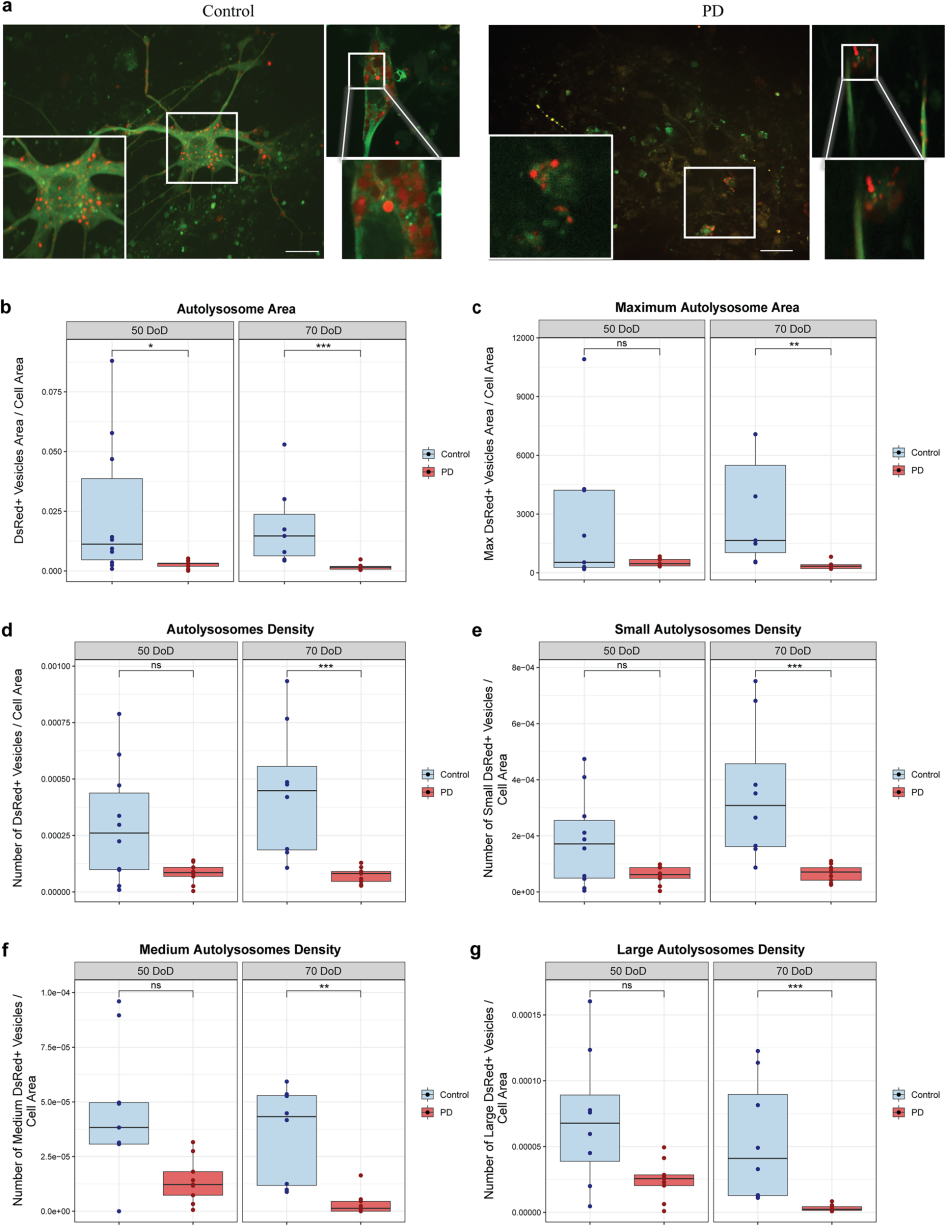
PD hMOs showed autophagy dysfunction. **a** Representative live-cell microscopy images of mosaic hMOs (55% LC3-Rosella expressing NESCs, 45% regular NESCs) at 70 DoD. Images show control and PD mosaic hMOs, with their respective zoomed region of interest. Scale bar: 20 μm. **b**−**g** Quantitative analysis of autophagy in hMOs using the LC3-Rosella reporter at 50 and 70 DoD. PD mosaic hMOs showed significant autophagy impairment in comparison to controls. **b** Total autolysosome area per cell area. **c** Maximum autolysosome area per cell area. **d** Number of autolysosomes per cell area. Number of small (**e**), medium (**f**), and large (**g**) autolysosomes per cell area. Data represent results from three independent experiments, with three fields per hMOs. Values are normalized to the average of controls across both DoD. Statistical analysis was performed using Wilcoxon *T*-test; *p < 0.05, **p < 0.01, and ***p < 0.001, with “ns” indicating non-significant results. Image analysis was performed using a custom MATLAB script for automated quantification of the different parameters of the autolysosome analysis.

For this, we assessed autophagy in mosaic hMOs at 50 and 70 DoD by live imaging. We focused on 50 and 70 DoD, as they represent intermediate and late stages of maturation, respectively. The schematic representation of hMOs generation is detailed in Supplementary Fig. 2. Representative images of control and PD mosaic hMOs from 70 DoD are shown in Fig. 2a, where PD organoids display a reduction in the autolysosomes.

Similar to neuronal cultures, we quantified the area and density of autolysosomes, categorizing them into small, medium, and large sizes based on their areas. Additionally, we analysed the maximum autolysosome area, representing the largest autolysosome present in each condition. Live imaging of Rosella-LC3 in mosaic hMOs showed that PD hMOs had decreased autolysosome area at 50 and 70 DoD (Fig. 2b). By 70 DoD, all autophagy parameters were reduced. PD hMOs presented reduced maximum autolysosome area, suggesting a possible dysfunction in the fusion or expansion of autolysosomes that are critical for proper cellular degradation (Fig. 2c). They also presented lower autolysosome density across all size categories when compared to controls, indicating a defect in the formation or maturation of autolysosomes at late stages of maturation (Fig. 2d-g).

To further characterize autophagy, we assessed LC3 and p62 flux by treating the hMOs with Bafilomycin A1 (BafA1) for 24 h. BafA1 is widely used for measuring the autophagic flux as it inhibits the fusion of autophagosomes with lysosomes. Thus, it allows us to measure the accumulation of LC3 and p62 markers^40^. Our results showed a decrease in LC3 and p62 flux at both time points in PD hMOs when compared to controls (Supplementary Fig. 3a-c). In addition, we quantified levels of LAMP2, a lysosomal membrane protein essential for autophagosome maturation and degradation. PD hMOs exhibited significantly reduced total LAMP2 levels compared to controls (Supplementary Fig. 3d), further supporting the presence of lysosomal dysfunction and impaired autophagic turnover. The consistency of reduced LC3-II, p62 flux, and LAMP2 levels across cellular models underscores the central role of lysosomal-autophagic impairment as a therapeutic target in PD.

### PD hMOs exhibit elevated levels of α-Synuclein and pS129

Building on our previous findings showing autophagy dysfunction in both PD models, we next investigated whether these impairments correlate with α-Synuclein accumulation and phosphorylation, two hallmark features of PD pathology.

**Fig. 3:**
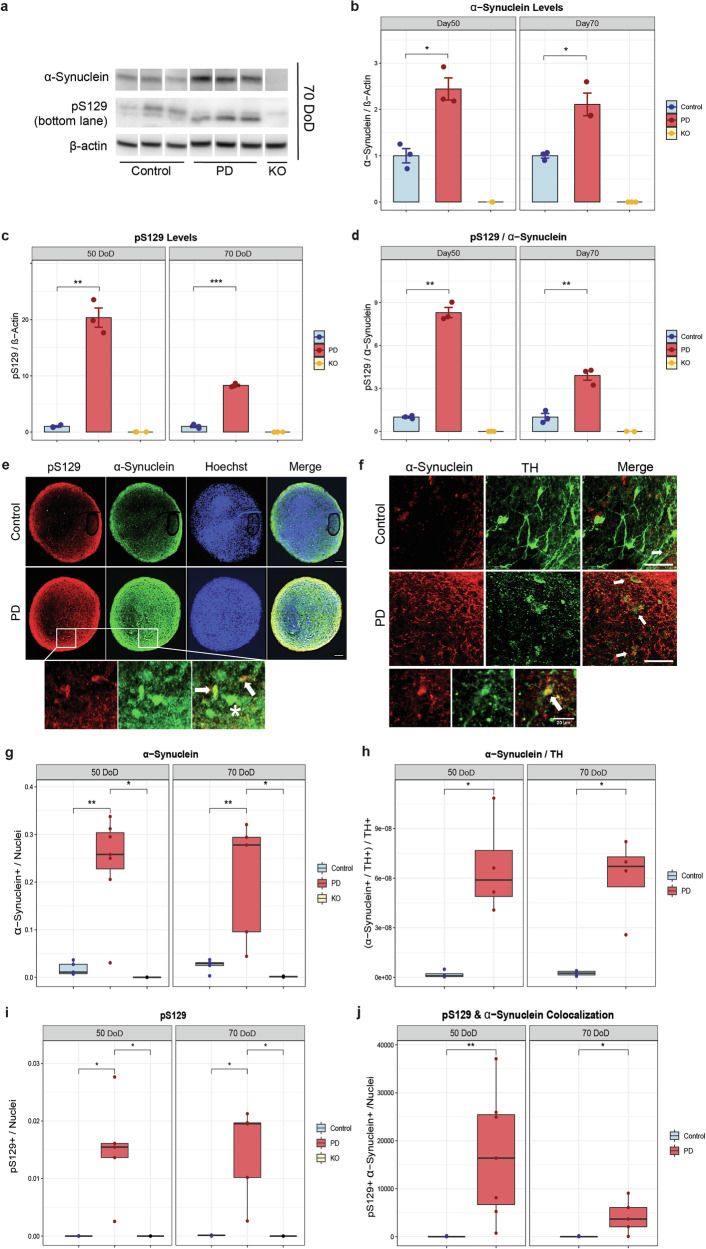
PD hMOs exhibit increased α-Synuclein and pS129 levels. **a** Representative Western blot images showing total α-Synuclein, pS129 and β-Actin in control, PD and *SNCA* knockout hMOs, which were used to validated the specificity of the antibodies for α-Synuclein and pS129. Total and pS129 α-Synuclein at 70 DoD were detected on separate Western blot membranes. The uncropped membranes of the blots shown in this figure are provided in Supplementary Figs 9-11. Bar graphs depict the protein levels of (**b**) total α-Synuclein, (**c**) pS129, and (**d**) the ratio of pS129 to total α-Synuclein where they are increased in PD hMOs. Protein expression was normalized to β-Actin. Error bars represent standard error of the mean (SEM). Immunoblot analysis was performed using ImageJ software. Data represent results from three independent experiments. Values are normalized to the average of controls at each time point. Statistical analysis was performed using Student’s *t*-test; *p < 0.05, with “ns” indicating non-significant results. **e**, **f** Representative immunofluorescence images of hMOs stained for pS129, total α-Synuclein, TH and nuclei. Arrows point to double-positive cells. **e** Asterisks (*) indicate cells positive for α-Synuclein but lacking pS129 signal, highlighting the diversity of α-Synuclein phosphorylation states. **f** Zoomed-in insets show regions of α-Synuclein colocalization within TH-positive neurons, emphasizing hallmark PD pathology. Scale bar: 200 μm. Quantification of (**g**) total α-Synuclein and (**h**) ratio of total α-Synuclein to TH, revealing accumulation of α-Synuclein in dopaminergic neurons in PD hMOs. Quantification of (**i**) pS129, and (**j**) colocalization of total and pS129 α-Synuclein, showing increased phosphorylation of α-Synuclein in PD hMOs. Data result from three independent experiments, each analysed from at least two hMOs replicates per condition. Values are normalized to the average of controls across all time points, allowing a comparison over time. Statistical analysis was performed using Wilcoxon *T*-test; *p < 0.05, **p < 0.01, with “ns” indicating non-significant results. Image analysis was performed using a custom MATLAB script for automated quantification of the different stainings. The absence of staining in *SNCA* knockout sections validated the specificity of the antibodies for α-Synuclein and pS129.

First, we investigated the levels of α-Synuclein, pS129 and the pS129/α-Synuclein ratio in hMOs at 50 and 70 DoD by Western Blot. Representative immunoblots are presented in Fig. 3a. PD hMOs showed increased levels of total α-Synuclein compared to controls at both time points (Fig. 3b). PD hMOs also had higher levels of pS129 and an increased pS129/α-Synuclein ratio (Fig. 3c, d).

To confirm and localize these findings at the cellular level, we performed immunofluorescence stainings. Representative images illustrated in Fig. 3e, f, show a widespread of α-Synuclein and pS129 in PD hMOs. The quantification confirmed that PD hMOs had more total α-Synuclein, particularly within TH-positive neurons (Fig. 3g, h), implying an accumulation of α-Synuclein in this neuronal population. Consistent with our Western Blot findings, immunofluorescence analysis also showed an increase in pS129 levels in PD hMOs (Fig. 3i). Staining specificity was confirmed by including *SNCA* knockout hMOs, where no detectable signal for pS129 or α-Synuclein was present (see Supplementary Fig. 4a). In addition, to further validate the specificity of the pS129 antibody, we performed the Lambda phosphatase assay. Lambda phosphatase treatment abolished pS129 immunoreactivity in both control and PD organoids, while total α‑Synuclein staining was preserved (Supplementary Fig. 4b, 4c). This assay was used only as a technical validation of the pS129 antibody and was not included in the main functional or phenotypic analyses. Both the pS129/α-Synuclein colocalization (Fig. 3j) and the ratio of pS129 to total α-Synuclein (see Supplementary Fig. 4d) were increased in PD hMOs.

The elevated levels of both total and pS129 α-Synuclein demonstrate that these models recapitulate key pathological features of PD.

### PD hMOs exhibit increased α-Synuclein aggregation

**Fig. 4:**
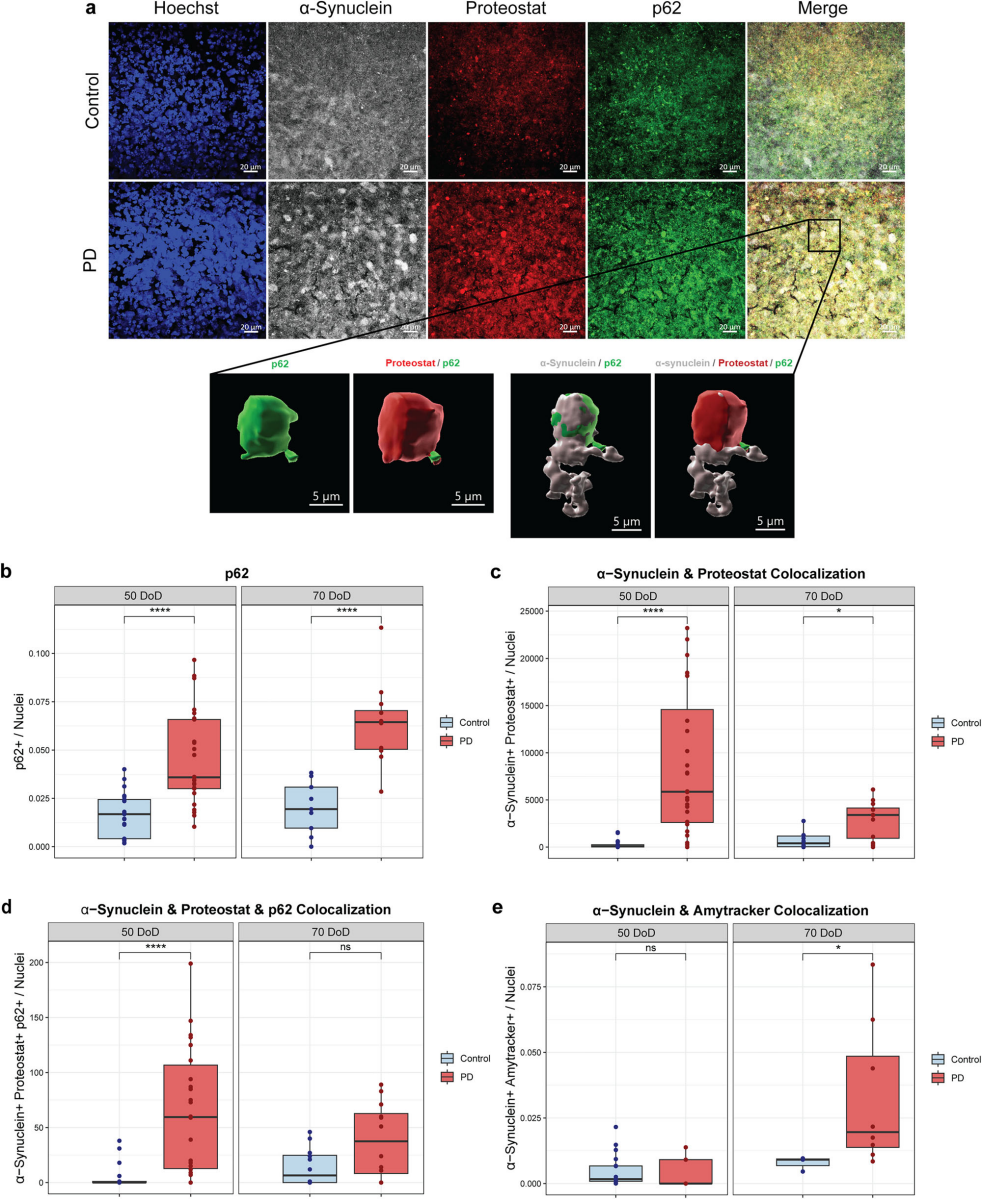
Increased α-Synuclein aggregation in PD hMOs. **a** Representative immunofluorescence images of hMOs stained for α-Synuclein (grey), p62 (green), Proteostat (red) and nuclei (blue) at 70 DoD as well as a 3D reconstruction. Scale bar: 5 and 20 μm. Quantification of (**b**) p62 immunoreactivity, (**c**) Colocalization of α-Synuclein and Proteostat, (**d**) triple colocalization between α-Synuclein, Proteostat, and p62 and (**e**) colocalization of α-Synuclein and AmyTracker in control and PD hMOs, at 50 and 70 DoD, where PD hMOs presented increased levels of aggregation markers. Data represent results from three independent experiments, each analysed from at least three hMOs replicates per condition. Values are normalized to the average of controls across all time points, allowing a comparison over time. Statistical analysis was performed using Wilcoxon *T*-test; *p < 0.05, **p < 0.01, ***p < 0.001, and ****p < 0.0001, with “ns” indicating non-significant results. Image analysis and colocalization quantification were performed using a custom MATLAB script for automated quantification.

Following our observation of elevated α-Synuclein and pS129 levels in PD hMOs and autophagy dysfunction, we investigated whether α-Synuclein aggregates are present in hMOs, at 50 and 70 DoD. Given that α-Synuclein aggregation is a hallmark of PD pathology, we performed immunostainings for markers of protein aggregation, including p62, Amytracker and Proteostat together with α-Synuclein, at 70 DoD (Fig. 4a). Lower magnification images can be found in Supplementary Fig. 4e. The Proteostat® Aggresome Detection Kit is a fluorescent dye that specifically binds to aggregated proteins, while the Amytracker 680 is a red dye that binds to protein aggregates with repetitive arrangement of β-sheets, labeling α-Synuclein aggregates.

In PD hMOs, p62 levels were elevated compared to controls at both times (Fig. 4b), which might indicate an accumulation of autophagosomes that are not properly fused with lysosomes. Besides being a marker of autophagy, p62 is a common component of protein aggregates in neurodegenerative diseases, including PD^41^, further confirming impaired clearance of protein aggregates. To specifically detect α-Synuclein aggregates, we performed colocalization analyses using the Proteostat and the Amytracker. We found that PD hMOs showed increased colocalization of α-Synuclein and Proteostat (Fig. 4c) as well as enhanced colocalization of α-Synuclein, Proteostat, and p62 (Fig. 4d), suggesting that while α-Synuclein is being targeted for autophagic clearance, it is not efficiently degraded. Moreover, we found higher colocalization between α-Synuclein and Amytracker in PD hMOs at 70 DoD (Fig. 4e), suggesting the presence of β-sheet-rich α-Synuclein aggregates, that are highly toxic and more resistant to degradation^42^.

### PD hMOs show dopaminergic neuronal degeneration and functional decline

**Fig. 5:**
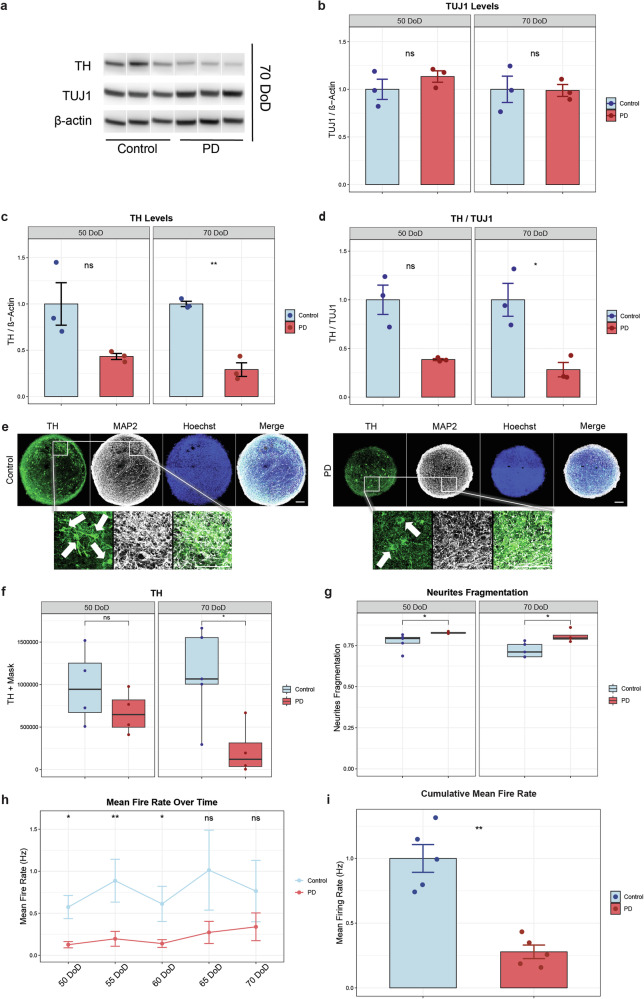
Progressive loss of dopaminergic characteristics and functional decline in PD hMOs. **a** Representative Western blot images showing TUJ1, TH, and β-Actin in control and PD hMOs. The uncropped membranes of the blots shown in this figure are provided in Supplementary Figs 9-11. β-actin was also used as housekeeping for data analysis shown in Fig. 3. Bar graphs depict protein levels of (**b**) TUJ1, (**c**) TH, and (**d**) the ratio of TH to TUJ1, showing decreased levels of TH in PD hMOs. Protein expression was normalized to β-Actin. Immunoblot analysis was performed using ImageJ software. Error bars represent SEM. Data represent results from three independent experiments. Values are normalized to the average of controls at each time point. Statistical analysis was performed using Wilcoxon *T*-test. **e** Representative immunofluorescence images of hMOs stained for MAP2 (red), TH (green) and nuclei (blue). Scale bar: 20 μm. Quantification of (**f**) TH-positive mask, (**g**) TH-positive neurites fragmentation, showing progressive dopaminergic neuron dysfunction and morphological changes in PD hMOs. Data represent results from three independent experiments, each analysed from at least two hMO replicates per condition. Values are normalized to the average of controls across all time points, allowing a comparison over time. Statistical analysis was performed using Wilcoxon *T*-test; *p < 0.05, with “ns” indicating non-significant results. **h** Mean fire rate of neurons in hMOs was measured every 5 days from 50 to 70 DoD. **i** Cumulative mean fire rate, showing decreased neuronal activity in PD hMOs. Data represent results from five independent experiments. Values are normalized to the average of controls at each time point. Statistical analysis was performed using Wilcoxon *T*-test; *p < 0.05, **p < 0.01, with “ns” indicating non-significant results. Error bars represent SEM. Image analysis was performed using MATLAB software for automated quantification of immunofluorescence staining.

Lastly, we investigated whether hMOs undergo dopaminergic neuron dysfunction, which may result from autophagy impairment and α-Synuclein accumulation. First, we analysed the levels of TH and TUJ1 (Neuron-specific class III beta-tubulin), which is a neuronal marker, by Western Blot. Representative immunoblots are illustrated in Fig. 5a. Western blot analysis at 50 and 70 DoD showed no significant difference in TUJ1 levels between PD and control hMOs (Fig. 5b) but decreased levels of TH and lower TH to TUJ1 ratio in PD hMOs at 70 DoD (Fig. 5c, d). Then, we performed immunostaining for further analysis, with representative images of MAP2 and TH at 70 DoD are shown in Fig. 5e. Immunostaining confirmed fewer dopaminergic neurons in PD hMOs at 70 DoD (Fig. 5f) and increased fragmentation of TH-positive neurites in PD hMOs at 50 and 70 DoD (Fig. 5g). This reduction in TH may reflect loss of dopaminergic phenotype, decreased TH expression, or structural changes in dopaminergic neurons, rather than absolute loss of dopaminergic cells, since the total neuronal population remained unchanged.

Finally, we used Microelectrode array (MEA) to assess neuronal network functional activity in hMOs. Electrophysiological activity was recorded from 50 to 70 DoD, with intervals of 5 days. PD hMOs showed a decrease in the mean fire rate over time as well as in the cumulative mean fire rate compared to controls (Fig. 5h, i). Moreover, PD hMOs also tend to have less bursting events and lower cumulative burst frequency compared to control hMOs (see Supplementary Fig. 5). In summary, these results indicate that in PD hMOs, loss of dopaminergic characteristics and functional decline emerge during late stages of culture. Importantly, these changes are specific to dopaminergic neurons, as general neuronal markers remained unchanged. Reduced electrophysiological activity in PD hMOs appears to precede changes in dopaminergic marker expression and structural integrity.

## Discussion

α-Synuclein aggregation and dopaminergic neuronal degeneration are the main neuropathological features of PD. Growing evidence suggest that autophagy dysregulation is a critical factor in PD pathology, particularly in the clearance of misfolded α-Synuclein. However, the timing and progression of autophagic processes are still poorly understood. This study explores the temporal dynamics of autophagy using live-cell imaging in two iPSCs-derived PD models, neuronal cultures and hMOs carrying the 3xSNCA mutation. Our results reveal that autophagy dysfunction emerges early in PD neuronal cultures, suggesting an early event in PD pathogenesis. In hMOs, while elevated α-Synuclein levels are already detectable at intermediate stages (50 DoD), full autophagy dysfunction becomes prominent only at later time points (70 DoD), supporting a context-dependent relationship between these pathological features.

Unlike traditional static analysis, the LC3-Rosella reporter system allows us to monitor autolysosomes in real time and therefore a better understanding of autophagy progression in PD, namely autophagosome maturation along with lysosome fusion. When integrating this strategy with biochemical analyses and with immunofluorescence analyses of α-Synuclein levels and dopaminergic markers, this strategy enabled a multidimensional perspective of disease progression.

Several studies have studied autophagy in iPSCs-derived dopaminergic neurons. In iPSCs-derived midbrain neurons carrying a 3xSNCA, LC3II and p62 flux was decreased and α-Synuclein impaired the autophagic flux shortly after autophagosome formation^43^. Moreover, iPSCs-derived neurons from *GBA1*-PD and *LRRK2*-PD with the G2019S mutation also exhibited autophagy impairment with decreased autophagic flux and accumulation of autophagosomes^44,45^. On the other hand, iPSCs-derived neurons carrying an *SNCA* triplication showed upregulated LC3II-autophagic flux, suggestive of induction of autophagy^46^. A few studies have also investigated the role of autophagy in hMOs. Impairments in the autophagy flux of hMOs carrying a N370S mutation in the *GBA* gene were observed, where these hMOs showed decreased LC3II/LC3I ratio and increased levels of p62^47^. Also, DJ1 KO hMOs revealed a decrease in the LC3II flux showing a failure in the autophagy^48^. Nevertheless, these studies were done in fixed samples, showing only a snapshot of a dynamic process. In our study, we assessed autophagy in live cells, providing a dynamic overview.

In PD neuronal cultures, autolysosome formation was reduced from the onset of differentiation. At 0 DoD, we observed a lower density of autolysosomes, especially of the small autolysosomes, compared to the control. Early in autophagy, an autolysosome grows as several lysosomes merge with an autophagosome^38^. However, persistent accumulation of large autolysosomes is often associated with impaired degradation capacity and reduced autophagic flux. On the other hand, smaller autolysosomes are associated with more efficient and active degradation^37,38^. As small autolysosomes are indicative of functional and active autophagic structures, these results suggest an early defect in autophagy initiation or autolysosome formation in PD neurons. At 3 DoD, we observed an increase in the autolysosome area in PD neurons accompanied by a decrease in the density of small autolysosomes but an increase in the density of large autolysosomes. Defects in the lysosome function also lead to an increase in the density of larger autolysosomes^49^. While evaluating lysosomal activity may provide additional insights into these findings, it falls outside the scope of the present study and should be addressed in future investigations. As differentiation continued, PD neurons exhibited diminished autolysosome area and density regardless of their size, indicating a severe blockage of this pathway. This impairment strengthens the idea that, as PD develops, autophagy is not only compromised early but declines further.

Similar results were observed in PD hMOs showing a significant autophagy impairment. At 50 DoD, PD hMOs had a decrease in the autolysosome area. This came before the prominent impairment that was seen at 70 DoD, when autolysosome density and area were considerably reduced across all size groups, corroborating our observations in PD neuronal cultures at late stages of differentiation. In addition to live-cell imaging, we included biochemical analyses of LC3-II and p62 following BafA1 treatment to measure autophagic flux as well the levels of LAMP2. These experiments confirmed a reduction in autophagic flux and decreased LAMP2 levels in PD hMOs, supporting the presence of impaired autophagy in our model (see Supplementary Fig. 3). Importantly, this autophagy dysfunction overlapped with increased accumulation of α-Synuclein, particularly in TH-positive neurons, in PD hMOs. Also, the increased ratio and colocalization of α-Synuclein with pS129 indicates the formation of aggregates. Mohamed et al. demonstrated that pS129 aggregates increased with the maturation of hMOs^32^. Although these observations point toward an altered phosphorylation profile of α-Synuclein in PD hMOs, further investigation is needed to understand the exact nature of these pS129-positive structures. While our data in PD hMOs clearly indicate impaired autophagic turnover, they do not allow us to determine the specific mechanistic step of impairment, such as induction, autophagosome-lysosome fusion, or lysosomal function. Further experiments will be necessary to pinpoint which aspect of the autophagy pathway is primarily affected in PD organoids.

To assess protein aggregation, we used additional markers such as p62, Proteostat, and Amytracker with α-Synuclein. Our results demonstrated elevated levels of p62 together with increased colocalization of α-Synuclein with Proteostat and Amytracker, and α-Synuclein with Proteostat and p62, confirming the presence of α-Synuclein aggregates resistant to degradation. While our analysis of p62, Proteostat, and α-Synuclein interactions was restricted to hMOs, previous studies have demonstrated p62 accumulation and α-Synuclein aggregation in neuronal cultures, supporting the relevance of these pathways in 2D systems as well^50^. It has been shown that α-Synuclein aggregates are resistant to degradation and inhibit autophagy by impairing autophagosome maturation^51^. Also, α-Synuclein fibrils have been found to alter lysosome morphology, reducing the clearance of aggregates by autophagy^52^. Although our results suggest a bidirectional link between autophagy and α-Synuclein levels, the autophagy system likely becomes overwhelmed with the increase of α-Synuclein levels.

Despite the use of a battery of aggregate-sensitive markers and Lambda phosphatase-based validation of pS129 antibody, we recognize that direct biochemical analyses, such as proteinase K digestion followed by western blotting, or filter trap assay, are necessary to conclusively confirm the insoluble, aggregated status of α-Synuclein in hMOs. Future studies should incorporate these approaches to provide definitive molecular evidence for protein aggregation in this model system.

We also explored how these impairments impact neuronal function. Neurons are particularly sensitive to changes in protein degradation pathways, with autophagy playing an important role in their survival^53^. A few studies have demonstrated that α-Synuclein can lead to early pathological synaptic alterations in PD, where disruptions in dopaminergic neurotransmission have been documented to precede Lewy pathology and neurodegeneration^54,55^. Thus, we assess the dopaminergic population and the electrophysiological activity of hMOs. In PD hMOs, a lower number of dopaminergic neurons at 70 DoD was observed. MEA analysis revealed that PD hMOs presented decreased neuronal activity. This functional decline correlated with autophagy dysfunction and α-Synuclein accumulation and aggregation, which altogether may be associated with dopaminergic dysfunction. These results are corroborated by previous studies from our group showing that accumulation of α-Synuclein and decreased electrophysiological activity precede dopaminergic degeneration in PD hMOs carrying an 3xSNCA^33,34^.

Although we report an interplay between α-Synuclein accumulation, autophagy dysfunction, and dopaminergic degeneration, additionally also other pathological processes are likely to be involved in PD progression. For instance, α-Synuclein oligomers triggered oxidative stress in iPSCs-derived neurons with a 3xSNCA^56^. Also, α-Synuclein can induce mitochondrial dysfunction, calcium homeostasis disorders, endoplasmic reticulum stress and neuroinflammation^57^. Exploring the role of these processes in autophagy in the context of 3xSNCA will provide a more thorough understanding of PD progression. Also, pharmacological or genetic rescue experiments would be necessary to directly test causality and to demonstrate whether restoring autophagic flux can alleviate α-Synuclein aggregation and neuronal loss.

While our study provides valuable insights, it naturally comes also with limitations. The 3xSNCA mutation represents a specific genetic form of PD, potentially limiting translation our findings to other genetic backgrounds. Additionally, our used hMO model, while more complex than neuronal cultures, still lacks critical cellular components, such as vascularization and immune components. For instance, the role of microglial autophagy has been described as a key factor in PD progression and pathogenesis^58^. Choi et al. demonstrated that microglia have a neuroprotective effect by participating in the clearance of α-Synuclein^59^. Moreover, longer studies in hMOs will provide a better understanding of late-stage PD pathology. Thus, the absence of functional vasculature, immune cells such as microglia, and full tissue maturation in our organoid model may limit the ability to fully recapitulate in vivo PD pathology, especially mechanisms involving neurovascular and neuroimmune interactions.

In conclusion, our study shows that autophagy dysfunction is a key characteristic of PD pathology. The integration of live-cell imaging and functional assays supports autophagy as a central player in PD pathogenesis and a promising target for early therapeutic intervention.

## Methods

### Ethical approval

This work with iPSCs has been approved by the Ethics Review Panel (ERP) of the University of Luxembourg and the national Luxembourgish Research Ethics Committee (CNER, Comité National d’Ethique de Recherche; CNER No. 201901/01).

### Generation and maintenance of iPSCs, NESCs, neuronal cultures, and hMOs

#### iPSCs culture

Five iPSCs lines were used in this study, including two healthy controls and three PD lines carrying the 3xSNCA. A Rosella construct paired with the protein LC3 was integrated into one healthy control line and one PD line for autophagy analysis, as previously described^35^ and in section LC3-Rosella Autophagy Reporter. The terms “Control-auto” and “PD-auto”, as named in Supplementary Table 1, refer to control or PD (3xSNCA) iPSC lines that were engineered to express the LC3-Rosella autophagy sensor. However, in this study and throughout the manuscript, all groups are classified as either Control or PD lines. Detailed information about the cell lines can be found in Supplementary Table 1. iPSCs were cultured in Essential 8 medium (ThermoFisher Scientific, cat. no. A1517001) supplemented with 1% Penicillin/Streptomycin (P/S, Invitrogen, cat. no. 15140122) on Matrigel-coated plates (Corning, cat. no. 354277). Cells were passaged every 3-4 days using Accutase (Sigma-Aldrich, cat. no. A6964). After splitting, the medium was supplemented with 10 μM ROCK inhibitor Y-27632 (Merck Millipore, cat. no. 688000) for 24 h to enhance cell survival. All cultures were maintained at 37 °C with 5% CO_2_.

#### NESCs culture

NESCs were derived from iPSCs as previously described by Reinhardt et al., (2013)^36^. The passage numbers of NESCs used for these experiments ranged from P10 to P14. NESCs were maintained in N2B27 medium on Matrigel-coated plates, supplemented with 0.75 μM purmorphamine (PMA; Enzo Life Sciences, cat. no. ALX-420-045), 3 μM CHIR-99021 (CHIR; Axon Medchem, cat. no. 1386), and 150 μM ascorbic acid (AA; Sigma-Aldrich, cat. no. A4403), referred to as N2B27 maintenance medium. N2B27 medium consisted of a 50:50 mixture of DMEM-F12 (Invitrogen; cat. no. 11320033) and Neurobasal (Invitrogen; cat. no. 21103049) with 1:200 N2 supplement (Invitrogen; cat. no. 17502048), 1:100 B27 supplement without Vitamin A (Invitrogen; cat. no. 12587010), 1% Glutamax (ThermoFisher Scientific, cat. no. 35050061), and 1% P/S. NESCs were passaged at 80–90% confluence using Accutase^29^. Once the quality of the derivation was validated, neuronal cultures and hMOs were generated.

#### Neuronal differentiation

For neuronal cultures, 3 × 10^4^ cells/well were seeded in Matrigel-coated cell carrier ultra plates (PerkinElmer, cat. no. 6055302). Neuronal differentiation was initiated by culturing NESCs in N2B27 maintenance medium for 2 days, followed by 6 days in N2B27 patterning medium supplemented with 200 μM AA, 500 μM Dibutyryl Cyclic Adenosine Monophosphate (db cAMP, Sigma-Aldrich, cat. no. D0627), 10 ng/mL human Glial cell line-derived Neurotrophic Factor (hGDNF, Peprotech, cat. no. 450-10), 10 ng/mL human Brain-Derived Neurotrophic Factor (hBDNF, Peprotech; cat. no. 450-02), 1 ng/mL Transforming growth factor beta 3 (TGF-β3; Peprotech; cat. no. 100-36E), and 1 μM PMA. The medium was changed every other day. Afterward, cells were cultured in N2B27 differentiation medium (identical to the patterning medium but without PMA) until the end of the experiments, totaling 11 DoD. Medium was changed every 3-4 days during this period. For the autophagy experiments, neuronal cultures were generated from NESCs containing the LC3-Rosella construct.

#### Organoid generation

hMOs were generated as previously described^29^, with slight modifications. A total of 9000 NESCs were seeded into each well of an ultra-low attachment 96-well round bottom plate (Corning, cat. no. 7007) and cultured in N2B27 maintenance medium for 2 days. For immunohistochemistry, western blot, and MEA analysis, hMOs were generated from regular NESCs. For the autophagy experiments, mosaic hMOs (55% LC3-Rosella NESCs + 45% regular) were generated to optimize signal resolution while reducing possible oversaturation, improving the autophagy measurements. The 3D colonies were then cultured in N2B27 patterning medium with medium changes every other day for 6 days. Differentiation was initiated with the N2B27 differentiation medium. To generate mosaic hMOs, the 3D colonies were embedded into 30 µl Geltrex droplets (Invitrogen, cat. no. A1413302), as previously described^29^, placed into 24-well ultra-low adhesion plates (Corning, cat. no. 3473), and kept at 37 °C and 5% CO_2_, under shaking conditions (80 rpm). For other experiments, 3D colonies were left in 96-well ultralow adhesion plates, at 37 °C and 5% CO_2_, in static conditions. All hMOs were kept in culture for 50 and 70 DoD, with medium changes every 3-4 days.

### Immunofluorescence of NESCs and neuronal cultures

Cells were fixed with 4% paraformaldehyde (PFA, Electron Microscopy Sciences, cat. no. 15710) in phosphate-buffered saline (PBS) for 15 min at room temperature (RT), followed by three washes with PBS for 5 min each. Permeabilization was performed using 0.5% Triton X-100 in PBS for 15 min at RT, followed by blocking with 10% fetal calf serum (FCS) in PBS for 1 h at RT. Cells were then incubated overnight at 4 °C with primary antibodies (Supplementary Table 2) diluted in 3% FCS in PBS. After three 5-min washes with PBS, cells were incubated with secondary antibodies (Supplementary Table 2) and Hoechst 33342 (1:10000, Life Technologies, cat. no. H3570) for nuclear staining, diluted in 3% FCS in PBS, for 1 h at RT in the dark. Three additional 5-min washes with PBS were performed.

For NESC characterization, cells were mounted with Fluoromount-G mounting (Southern Biotech, cat. no. 0100-01) and imaged using a Zeiss LSM 710 Confocal Inverted Microscope (*RRID:SCR_018063*) with a total magnification of 200x.

For other stainings, cell carrier ultra plates with duplicates per condition from three independent experiments were imaged in an automated manner using the Yokogawa CellVoyager CV8000 microscope (*RRID:SCR_023270*) with a 20x objective.

### Immunofluorescence of hMOs

hMOs were fixed overnight with 4% PFA in PBS at 4 °C and washed three times with PBS for 15 min each at RT. One hMO per condition and independent experiment was embedded in 3% low-melting point agarose (Sigma-Aldrich, cat. no. A9414) in PBS. The agarose block was sectioned using a vibrating blade microtome (Leica VT1000s, *RRID:SCR_016495*) into 50 µm sections that were collected sequentially into three wells of a 48-well plate. Sections were permeabilized and blocked for 2 h at RT in blocking buffer (2.5% normal goat or donkey serum, 2.5% bovine serum albumin, 0.1% sodium azide, and 0.5% Triton X-100 in PBS) with shaking. Sections were then incubated with primary antibodies (Supplementary Table 2) diluted in blocking buffer containing 0.1% Triton X-100, for 48 h at 4 °C with gentle shaking. Then, sections were washed 3x with 0.01% Triton X-100 in PBS for 10 min each and subsequently incubated with the secondary antibodies (Supplementary Table 2), along with Hoechst 33342 (1:10000 dilution), diluted in blocking buffer containing 0.1% Triton X-100, for 2 h at RT with shaking. Sections were washed three times with 0.01% Triton X-100 in PBS for 10 min each and once with Milli-Q water. For protein aggregates, we used the Proteostat® Aggresome Detection Kit (Enzo, cat no. ENZ-51035) and Amytracker 680 (Ebba Biotech AB) protocols. Any remaining agarose was carefully removed before mounting the sections with Fluoromount-G mounting medium. Sequential collection and staining of sections were implemented to avoid bias in selecting specific hMO regions.

Imaging of the sections from at least three independent experiments, with at least two hMOs replicates per condition was performed using a Yokogawa CellVoyager CV8000 high-content screening microscope. For α-Synuclein aggregates detection, z-planes were acquired using a 60x oil objective. For other high-content image analyses, z-planes were imaged with a 20x objective.

### Lambda phosphatase validation of pS129

For specific validation of the pS129 antibody, hMOs were generated from midbrain floor plate neural progenitor cells (mfNPCs) derived from iPSCs, using a reduced seeding density of 6000 cells, following previously established protocols^47,60^. In addition to the core control and PD iPSC lines used throughout this study, two further control lines and one PD line were included to increase the robustness of this antibody‑validation assay across multiple genetic backgrounds and were used exclusively for the Lambda phosphatase experiment (Supplementary Table S3).

hMOs were fixed overnight in 4% PFA at 4 °C, washed in PBS and sectioned at 70 µm. Then, hMO sections were incubated with 400 U Lambda phosphatase in 1x Lambda phosphatase buffer supplemented with 1x MnCl₂ (2 mM) (Santa Cruz, cat. no. sc‑200312 A) for 30 min at 37 °C, or with reaction buffer lacking enzyme for control conditions. After treatment, sections were washed and subjected to immunostaining for total α‑Synuclein and pS129 using the same protocol as described above.

### LC3-Rosella autophagy reporter

The LC3-Rosella construct, which combines the pH-sensitive green fluorescent protein pHluorin and the red fluorescent protein from Discosoma (DsRed) with LC3II, was integrated into iPSCs lines via nucleofection, using the P3 Primary Cell 4D-Nucleofector® X Kit L (Lonza Bioscience, cat. no. V4XP-3024), as previously described^35,61^. Transfected iPSCs were sorted by fluorescence-activated cell sorting (FACS) to enrich positive clones and screened by fluorescence microscopy to confirm LC3-Rosella expression. The use of pH-sensitive fluorescent proteins enables the distinction of several subcellular compartments. This happens because the pHluorin signal, a modified form of GFP, is suppressed in acidic conditions, such as the interior of the autolysosome, while DsRed continues to fluorescence. However, since LC3 is a main component of the neuronal cytoskeleton^62^, pHluorin signal is very diffuse in neuronal cultures and hMOs. Hence, our analysis focused on autolysosomes identified as red-only vesicles (pHIuorin-DsRed+ structures). For imaging, pHluorin and DsRed were excited using 488 nm and 561 nm lasers, respectively.

### Live imaging in neuronal cultures

Autophagy dynamics were monitored in live neuronal cultures at 0, 3, 5, 7, 9, and 11 DoD using the Yokogawa CV8000 microscope, under controlled atmosphere conditions (37 °C and 5% CO_2_). Cell carrier ultra plates with triplicates per condition from three independent experiments were imaged, acquiring a total of 25 fields per well. DsRed and pHluorin images were acquired in parallel using camera binning 2 and a 20x objective. Cells were kept under normal conditions between imaging sessions.

### Live imaging of hMOs

Live imaging of LC3-Rosella in hMOs was performed at 50 and 70 DoD using the Zeiss Cell Observer Spinning Disk confocal microscope, under controlled atmosphere conditions (37 °C and 5% CO_2_). Individual hMOs from three independent experiments were transferred to a µ-Slide 8 Well Chambered Coverslip (Ibidi, cat. no. 80826) and allowed to attach for 45 min without medium in the incubator. Then, N2B27 maturation medium was added, and hMOs were imaged live. DsRed and pHluorin signals were imaged in parallel using a 40x oil objective. A total of three fields per hMO were acquired. After imaging, hMOs were then returned to culture for further analysis.

### Image processing and autolysosome classification

Images of neuronal cultures and hMOs were processed and analysed using custom algorithms developed in MATLAB (2020a, Mathworks; *RRID:SCR_001622*), as described by our group^29,35^. For dual and triple marker colocalization, we used custom MATLAB scripts. Each fluorescence channel was processed to create binary masks for the markers of interest, using intensity thresholding. Colocalization was identified where masks overlapped within each image plane. The percentage of pixels that were double- or triple-positive was calculated in each plane and used for quantification. All analysis steps were standardized across samples. These in-house imaging analysis pipelines allow an automated identification and segmentation of cellular structures, such as nuclei, neurons and autophagosomes by extracting their specific features.

For the autophagy experiments, we performed a broad size classification of autolysosomes based on voxel area, derived from histogram analysis of size distribution for each model. This size-based classification allowed us to do a more detailed analysis of autophagy in each condition. In neuronal cultures, autolysosomes were defined as small (<8 voxels, corresponding to <0.09 µm²), medium (8 to 25 voxels, corresponding to 0.09-0.29 µm²), and large (≥25 voxels, corresponding to ≥0.29 µm²). In mosaic hMOs, given their greater structural complexity and signal distribution, we adjusted the thresholds to small (<25 voxels, <0.29 µm²), medium (25 to 80 voxels, 0.29-0.93 µm²), and large (≥80 voxels, ≥0.93 µm²). These µm² values were calculated based on the voxel dimensions from the imaging system. The maximum autolysosome area was defined as the single largest autolysosome, in terms of area, identified per imaged field, and was calculated through automated segmentation and analysis in our MATLAB pipeline.

### Western blot

Five hMOs per condition were lysed using RIPA buffer (Abcam, cat no. ab156034) supplemented with protease inhibitor cocktail (Roche, cat no. 11697498001) and phosphatase inhibitor cocktail (Merck Millipore, cat no. 524629). To improve protein extraction and sample quality, lysates were sonicated for 10 cycles (30 s on/30 s off) using the Bioruptor Pico (Diagenode) and centrifuged at 14,000 x g for 20 min at 4 °C. Protein concentrations were determined using the Pierce™ BCA Protein Assay Kit (ThermoFisher Scientific, cat no. 23225). Protein samples (10 µg) were prepared and boiled at 95 °C for 5 min in loading buffer. Proteins were separated on 4-12% NuPAGE™ Bis-Tris polyacrylamide gels (ThermoFisher Scientific), using MES SDS running buffer (ThermoFisher Scientific), at 110 V for 1 h 40 min. Proteins were transferred to polyvinylidene difluoride (PVDF) membranes using the iBlot™ 2 Gel Transfer Device (ThermoFisher Scientific), following the manufacturer’s instructions. Membranes were fixed in 0.04% PFA for 30 min at RT and washed three times with 0.01% Tween in PBS (PBS-T) for 5 min each. Then, membranes were blocked in 5% non-fat powdered milk (Roth, cat. no. T145.2) prepared in 0.02% PBS-T for 1 h at RT, followed by overnight incubation at 4 °C with primary antibodies (Supplementary Table 2) diluted in 5% BSA in 0.02% PBS-T. After three 5-min washes with 0.01% PBS-T, membranes were incubated with HRP-linked secondary antibodies (anti-rabbit IgG (H + L) or anti-mouse IgG (H + L), VWR) at a 1:1000 dilution in 0.02% PBS-T for 2 h at RT. Membranes were then washed three times with 0.01% PBS-T 5 min each, and protein bands were detected using SuperSignal West Pico Chemiluminescent Substrate (ThermoFisher Scientific, cat. no. 34580) and imaged with the STELLA 8300 imaging system (Raytest). Image analysis was performed using ImageJ software (NIH; *RRID:SCR_003070*). Some membranes were stripped using Restore™ Western Blot Stripping Buffer (Thermo Fisher Scientific, cat. no. 21063) for 5 min at RT, according to the manufacturer’s instructions. Band intensities were normalized to β-actin as a loading control. Samples from the same independent experiment were loaded onto a single gel, and all blots were processed using the same experimental conditions. Western blot experiments were conducted from 3 independent experiments per condition. Unprocessed membranes of all blots are provided in Supplementary Figs. 6-11, together with molecular weight markers.

### MEA analysis

MEA recordings were performed using the Maestro system (Axion BioSystems) to record the spontaneous electrical activity of hMOs, from 50 to 70 DoD, with intervals of 5 days. Before the recordings, 48-well MEA plates (16 electrodes per well) were coated with 0.1 mg/mL poly-D-lysine hydrobromide (Sigma, cat. no. P7886) and 10 μg/mL laminin (Sigma, cat. no. L2020) at 37 °C. Then, 45-day-old hMOs were carefully positioned onto the electrode arrays and secured with a droplet of Geltrex (Invitrogen, cat. no. A1413302). Following a short polymerization period, N2B27 differentiation medium was added. 24 h before each recording session, fresh N2B27 differentiation medium was added to the hMOs.

MEA recordings were performed as previously described^29,34,63^. Spontaneous activity was recorded for 10 minutes at 37 °C. Data acquisition and analysis were performed using Axion Integrated Studio (AxIS 2.1; *RRID:SCR_016308*) software. To minimize false positives and missed detections, a Butterworth band-pass filter (200-3000 Hz) and a threshold of 6x standard deviation were applied. Electrodes registering an average of ≥5 spikes/min were considered active. Neural stat compiler files were used for data analysis in MATLAB. The parameters analysed were spike frequency and burst analysis. This non-invasive approach allowed for longitudinal monitoring of hMOs maturation and network development over time. For each condition, hMOs were analysed in each of the five independent experiments.

### Statistical analysis

Data process and statistical analyses were performed using R software (R version 4.3.0 - “Already Tomorrow”, *RRID:SCR_001905*). For each cell line and experiment, data were grouped by condition, with mean values calculated. All experiments were performed with a minimum of three independent experiments and at least two technical replicates per condition unless otherwise stated. We recently showed that hMOs data are reproducible across batches and reliably capture PD disease phenotypes, supporting the robustness of our current experimental and quantification strategy^64^. The normality test was done by using the Shapiro-Wilk test. Statistical comparisons between control and experimental groups (Control vs. PD) were conducted using the Wilcoxon signed-rank test or *t*-test, depending on data distribution. Significance levels are denoted as *p* < 0.05 (*), *p* < 0.01 (**), *p* < 0.001 (***), and *p* < 0.0001 (****), with “ns” indicating non-significant results. Plots were generated using R Studio, and sample sizes, replicates, and experimental batches are detailed in figure legends.

## Supplementary information

Supplementary Information

## Data Availability

All data generated or analysed during this study as well as scripts that support the findings are available at 10.17881/gd2k-9685.

## Code availability

All scripts used to obtain, analyse and plot the data are available at https://gitlab.com/uniluxembourg/lcsb/developmental-and-cellular-biology/almeida_2025.

## References

[1] Maroteaux, L., Campanelli, J. T. & Scheller, R. H. Synuclein: A neuron-specific protein localized to the nucleus and presynaptic nerve terminal. *J. Neurosci.* **8**, 2804–2815 (1988). 3411354 10.1523/JNEUROSCI.08-08-02804.1988 PMC6569395

[2] Gao, V., Briano, J. A., Komer, L. E. & Burré, J. Functional and Pathological Effects of α-Synuclein on Synaptic SNARE Complexes. *J. Mol. Biol.* **435**, 167714 (2023). 35787839 10.1016/j.jmb.2022.167714 PMC10472340

[3] Polymeropoulos, M. H. et al. Mutation in the α-synuclein gene identified in families with Parkinson’s disease. *Science (80-)* **276**, 2045–2047 (1997). 10.1126/science.276.5321.2045 9197268

[4] Krüger, R. et al. Ala30Pro mutation in the gene encoding alpha-synuclein in Parkinson’s disease. *Nat. Genet.* **18**, 106–108 (1998). 9462735 10.1038/ng0298-106

[5] Zarranz, J. J. et al. The New Mutation, E46K, of α-Synuclein Causes Parkinson and Lewy Body Dementia. *Ann. Neurol.* **55**, 164–173 (2004). 14755719 10.1002/ana.10795

[6] Ibáñez, P. et al. Causal relation between alpha-synuclein gene duplication and familial Parkinson’s disease. *Lancet* **364**, 1169–1171 (2004). 15451225 10.1016/S0140-6736(04)17104-3

[7] Singleton, A. B. et al. α-Synuclein Locus Triplication Causes Parkinson’s Disease. *Science (80-)* **302**, 841 (2003). 10.1126/science.1090278 14593171

[8] Ferese, R. et al. Four Copies of SNCA Responsible for Autosomal Dominant Parkinson’s Disease in Two Italian Siblings. *Parkinsons. Dis.* **2015**, 546462 (2015). 26635992 10.1155/2015/546462 PMC4655296

[9] Farrer, M. et al. Comparison of Kindreds with Parkinsonism and α-Synuclein Genomic Multiplications. *Ann. Neurol.* **55**, 174–179 (2004). 14755720 10.1002/ana.10846

[10] Uversky, V. N., Li, J. & Fink, A. L. Evidence for a Partially Folded Intermediate in α-Synuclein Fibril Formation. *J. Biol. Chem.* **276**, 10737–10744 (2001). 11152691 10.1074/jbc.M010907200

[11] Calabresi, P. et al. Alpha-synuclein in Parkinson’s disease and other synucleinopathies: from overt neurodegeneration back to early synaptic dysfunction. *Cell Death Dis* **14**, 176 (2023). 36859484 10.1038/s41419-023-05672-9 PMC9977911

[12] Levine, B. & Kroemer, G. uido Autophagy in the Pathogenesis of Disease. *Cell* **132**, 27–42 (2008). 18191218 10.1016/j.cell.2007.12.018 PMC2696814

[13] Sahoo, S., Padhy, A. A., Kumari, V. & Mishra, P. Role of Ubiquitin–Proteasome and Autophagy-Lysosome Pathways in α-Synuclein Aggregate Clearance. *Mol. Neurobiol.* **59**, 5379–5407 (2022). 35699874 10.1007/s12035-022-02897-1

[14] Majeski, A. E. & Fred Dice, J. Mechanisms of chaperone-mediated autophagy. *Int. J. Biochem. Cell Biol.* **36**, 2435–2444 (2004). 15325583 10.1016/j.biocel.2004.02.013

[15] Xilouri, M., Vogiatzi, T., Vekrellis, K., Park, D. & Stefanis, L. Abberant α-synuclein confers toxicity to neurons in part through inhibition of chaperone-mediated autophagy. *PLoS One* **4**, 16–20 (2009). 10.1371/journal.pone.0005515 PMC2677735 19436756

[16] Cuervo, A. M., Stafanis, L., Fredenburg, R., Lansbury, P. T. & Sulzer, D. Impaired degradation of mutant α-synuclein by chaperone-mediated autophagy. *Science (80-)* **305**, 1292–1295 (2004). 10.1126/science.1101738 15333840

[17] Bloem, B. R., Okun, M. S. & Klein, C. Parkinson’s disease. *Lancet* **397**, 2284–2303 (2021). 33848468 10.1016/S0140-6736(21)00218-X

[18] Dehay, B. et al. Pathogenic lysosomal depletion in Parkinson’s disease. *J. Neurosci.* **30**, 12535–12544 (2010). 20844148 10.1523/JNEUROSCI.1920-10.2010 PMC6633458

[19] Cooper, J. M. et al. Chaperone-Mediated Autophagy Markers in Parkinson Disease Brains. *Arch. Neurol.* **67**, 1464–1472 (2010). 20697033 10.1001/archneurol.2010.198

[20] Xilouri, M. et al. Impairment of chaperone-mediated autophagy induces dopaminergic neurodegeneration in rats. *Autophagy* **12**, 2230–2247 (2016). 27541985 10.1080/15548627.2016.1214777 PMC5103347

[21] Laperle, A. H. et al. iPSC modeling of young-onset Parkinson’s disease reveals a molecular signature of disease and novel therapeutic candidates. *Nat. Med.* **26**, 289–299 (2020). 31988461 10.1038/s41591-019-0739-1

[22] Mazzulli, J. R., Zunke, F., Isacson, O., Studer, L. & Krainc, D. -Synuclein-induced lysosomal dysfunction occurs through disruptions in protein trafficking in human midbrain synucleinopathy models. *Proc. Natl. Acad. Sci. USA.* **113**, 1931–1936 (2016). 26839413 10.1073/pnas.1520335113 PMC4763774

[23] Bose, A., Petsko, G. A. & Studer, L. Induced pluripotent stem cells: a tool for modeling Parkinson’s disease. *Trends Neurosci* **45**, 608–620 (2022). 35667922 10.1016/j.tins.2022.05.001 PMC9576003

[24] Zahumenska, R. et al. Induced pluripotency: A powerful tool for in vitro modeling. *Int. J. Mol. Sci.* **21**, 1–19 (2020). 10.3390/ijms21238910 PMC7727808 33255453

[25] Bhargava, A., Sandoval Castellanos, A. M., Shah, S. & Ning, K. An insight into the iPSCs-derived two-dimensional culture and three-dimensional organoid models for neurodegenerative disorders. *Interface Focus* **12**, 20220040 (2022). 35992771 10.1098/rsfs.2022.0040 PMC9372641

[26] Robinton, D. A. & Daley, G. Q. The promise of induced pluripotent stem cells in research and therapy. *Nature* **481**, 295–305 (2012). 22258608 10.1038/nature10761 PMC3652331

[27] De Souza, N. Organoids. *Nat. Methods* **15**, 23 (2018).

[28] Jo, J. et al. Midbrain-like Organoids from Human Pluripotent Stem Cells Contain Functional Dopaminergic and Neuromelanin-Producing Neurons. *Cell Stem Cell* **19**, 248–257 (2016). 27476966 10.1016/j.stem.2016.07.005 PMC5510242

[29] Monzel, A. S. et al. Derivation of Human Midbrain-Specific Organoids from Neuroepithelial Stem Cells. *Stem Cell Reports* **8**, 1144–1154 (2017). 28416282 10.1016/j.stemcr.2017.03.010 PMC5425618

[30] Hu, X. et al. Modeling Parkinson’s Disease Using Induced Pluripotent Stem Cells. *Stem Cells Int* **2020**, 1–15 (2020). 10.1155/2020/1061470 PMC7091557 32256606

[31] Jo, J. et al. Lewy Body–like Inclusions in Human Midbrain Organoids Carrying Glucocerebrosidase and α-Synuclein Mutations. *Ann. Neurol.* **90**, 490–505 (2021). 34288055 10.1002/ana.26166 PMC9543721

[32] Mohamed, N. V. et al. Midbrain organoids with an SNCA gene triplication model key features of synucleinopathy. *Brain Commun* **3**, 1–21 (2021). 10.1093/braincomms/fcab223 PMC8495137 34632384

[33] Muwanigwa, M. N. et al. Alpha-synuclein pathology is associated with astrocyte senescence in a midbrain organoid model of familial Parkinson’s disease. *Mol. Cell. Neurosci.* **128**, 103919 (2024). 38307302 10.1016/j.mcn.2024.103919

[34] Modamio, J. et al. Synaptic decline precedes dopaminergic neuronal loss in human midbrain organoids harboring a triplication of the SNCA gene. *bioRxiv* (2021) 10.1101/2021.07.15.452499.

[35] Arias-Fuenzalida, J. et al. Automated high-throughput high-content autophagy and mitophagy analysis platform. *Sci. Rep.* **9**, 9455 (2019). 31263238 10.1038/s41598-019-45917-2 PMC6603000

[36] Reinhardt, P. et al. Derivation and Expansion Using Only Small Molecules of Human Neural Progenitors for Neurodegenerative Disease Modeling. *PLoS One* **8**, e59252 (2013). 23533608 10.1371/journal.pone.0059252 PMC3606479

[37] Kim, S., Choi, S. & Kang, D. Quantitative and qualitative analysis of autophagy flux using imaging. *BMB Rep* **53**, 241–247 (2020). 32317089 10.5483/BMBRep.2020.53.5.046 PMC7262512

[38] Su, Q. P. et al. Vesicle Size Regulates Nanotube Formation in the Cell. *Sci. Rep.* **6**, 1–11 (2016). 27052881 10.1038/srep24002 PMC4823757

[39] Smits, L. M. et al. Modeling Parkinson ’ s disease in midbrain-like organoids. *npj Park. Dis*. (2019) 10.1038/s41531-019-0078-4. 10.1038/s41531-019-0078-4 PMC6450999 30963107

[40] Mauvezin, C. & Neufeld, T. P. Bafilomycin A1 disrupts autophagic flux by inhibiting both V-ATPase-dependent acidification and Ca-P60A/SERCA-dependent autophagosome-lysosome fusion. *Autophagy* **11**, 1437–1438 (2015). 26156798 10.1080/15548627.2015.1066957 PMC4590655

[41] Bjørkøy, G. et al. p62/SQSTM1 forms protein aggregates degraded by autophagy and has a protective effect on huntingtin-induced cell death. *J. Cell Biol.* **171**, 603–614 (2005). 16286508 10.1083/jcb.200507002 PMC2171557

[42] Emin, D. et al. Small soluble α-synuclein aggregates are the toxic species in Parkinson’s disease. *Nat. Commun.* **13**, 1–15 (2022). 36127374 10.1038/s41467-022-33252-6 PMC9489799

[43] Pitcairn, C., Murata, N., Zalon, A. J., Stojkovska, I. & Mazzulli, J. R. Impaired Autophagic-Lysosomal Fusion in Parkinson’s Patient Midbrain Neurons Occurs through Loss of ykt6 and Is Rescued by Farnesyltransferase Inhibition. *J. Neurosci.* **43**, 2615–2629 (2023). 36788031 10.1523/JNEUROSCI.0610-22.2023 PMC10082462

[44] Schöndorf, D. C. et al. IPSC-derived neurons from GBA1-associated Parkinson’s disease patients show autophagic defects and impaired calcium homeostasis. *Nat. Commun*. **5**, (2014). 10.1038/ncomms5028 24905578

[45] Sánchez-Danés, A. et al. Disease-specific phenotypes in dopamine neurons from human iPS-based models of genetic and sporadic Parkinson’s disease. *EMBO Mol. Med.* **4**, 380–395 (2012). 22407749 10.1002/emmm.201200215 PMC3403296

[46] Oliveira, L. M. A. et al. Elevated α-synuclein caused by SNCA gene triplication impairs neuronal differentiation and maturation in Parkinson’s patient-derived induced pluripotent stem cells. *Cell Death Dis* **6**, 1–13 (2015). 10.1038/cddis.2015.318 PMC4670926 26610207

[47] Rosety, I. et al. Impaired neuron differentiation in GBA-associated Parkinson’s disease is linked to cell cycle defects in organoids. *npj Park. Dis.* **9**, 1–16 (2023). 10.1038/s41531-023-00616-8 PMC10728202 38110400

[48] Morrone Parfitt, G. et al. Disruption of lysosomal proteolysis in astrocytes facilitates midbrain organoid proteostasis failure in an early-onset Parkinson’s disease model. *Nat. Commun.* **15**, 447 (2024). 38200091 10.1038/s41467-024-44732-2 PMC10781970

[49] Mauvezin, C., Nagy, P., Juhász, G. & Neufeld, T. P. Autophagosome-lysosome fusion is independent of V-ATPase-mediated acidification. *Nat. Commun.* **6**, 7007 (2015). 25959678 10.1038/ncomms8007 PMC4428688

[50] Leupold, L., Sigutova, V., Gerasimova, E. & Regensburger, M. The Quest for Anti- α -Synuclein Antibody Specificity — Lessons Learnt From Flow Cytometry Analysis. **13**, 1–18 (2022). 10.3389/fneur.2022.869103 PMC9334871 35911883

[51] Tanik, S. A., Schultheiss, C. E., Volpicelli-Daley, L. A., Brunden, K. R. & Lee, V. M. Y. Lewy body-like α-synuclein aggregates resist degradation and impair macroautophagy. *J. Biol. Chem.* **288**, 15194–15210 (2013). 23532841 10.1074/jbc.M113.457408 PMC3663539

[52] Senol, A. D. et al. α-Synuclein fibrils subvert lysosome structure and function for the propagation of protein misfolding between cells through tunneling nanotubes. *PLoS Biol* **19**, e3001287 (2021). 34283825 10.1371/journal.pbio.3001287 PMC8291706

[53] Dehay, B. et al. Lysosomal impairment in Parkinson’s disease. *Mov. Disord.* **28**, 725–732 (2013). 23580333 10.1002/mds.25462 PMC5131721

[54] Janezic, S. et al. Deficits in dopaminergic transmission precede neuron loss and dysfunction in a new Parkinson model. *Proc. Natl. Acad. Sci. USA.* **110**, E4016–E4025 (2013). 24082145 10.1073/pnas.1309143110 PMC3801069

[55] Milber, J. M. et al. Parkinson disease and incidental Lewy body disease. *Neurology* **85**, 1670–1679 (2015). 26468408 10.1212/WNL.0000000000002102 PMC4653112

[56] Deas, E. et al. Alpha-synuclein oligomers interact with metal ions to induce oxidative stress and neuronal death in Parkinson’s disease. *Antioxidants Redox Signal* **24**, 376–391 (2016). 10.1089/ars.2015.6343 PMC4999647 26564470

[57] Lv, Q. K. et al. Role of α-synuclein in microglia: autophagy and phagocytosis balance neuroinflammation in Parkinson’s disease. *Inflamm. Res.* **72**, 443–462 (2023). 36598534 10.1007/s00011-022-01676-x

[58] Zhu, R., Luo, Y., Li, S. & Wang, Z. The role of microglial autophagy in Parkinson’s disease. *Front. Aging Neurosci.* **14**, 1039780 (2022). 36389074 10.3389/fnagi.2022.1039780 PMC9664157

[59] Choi, I. et al. Microglia clear neuron-released α-synuclein via selective autophagy and prevent neurodegeneration. *Nat. Commun.* **11**, 1386 (2020). 32170061 10.1038/s41467-020-15119-w PMC7069981

[60] Nickels, S. L. et al. Reproducible generation of human midbrain organoids for in vitro modeling of Parkinson’s disease. *Stem Cell Res* **46**, 101870 (2020). 32534166 10.1016/j.scr.2020.101870

[61] Sargsyan, A. et al. Rapid parallel measurements of macroautophagy and mitophagy in mammalian cells using a single fluorescent biosensor. *Sci. Rep.* **5**, 1–11 (2015). 10.1038/srep12397 PMC4517063 26215030

[62] Kouno, T. et al. Solution structure of microtubule-associated protein light chain 3 and identification of its functional subdomains. *J. Biol. Chem.* **280**, 24610–24617 (2005). 15857831 10.1074/jbc.M413565200

[63] Barmpa, K. et al. Modeling early phenotypes of Parkinson’s disease by age-induced midbrain-striatum assembloids. *Commun. Biol.* **7**, 1561 (2024). 39580573 10.1038/s42003-024-07273-4 PMC11585662

[64] Zuccoli, E. et al. Reproducibility of PD patient-specific midbrain organoid data for in vitro disease modeling. *iScience* **28**, (2025). 10.1016/j.isci.2025.113541 PMC12494858 41050933

