# Supplementary material for: Autophagy dysfunction in iPSCs-derived neurons and midbrain organoids carrying a SNCA triplication
Source: NPJ Parkinsons Dis. 2026 Mar 31;12:123. doi: 10.1038/s41531-026-01330-x (PMC13199463; doi:10.1038/s41531-026-01330-x)
Supplement: Supplementary file 1 — Supplementary Information [file 41531_2026_1330_MOESM1_ESM.docx]

**Supplementary Data**

***Supplementary Figure 1***


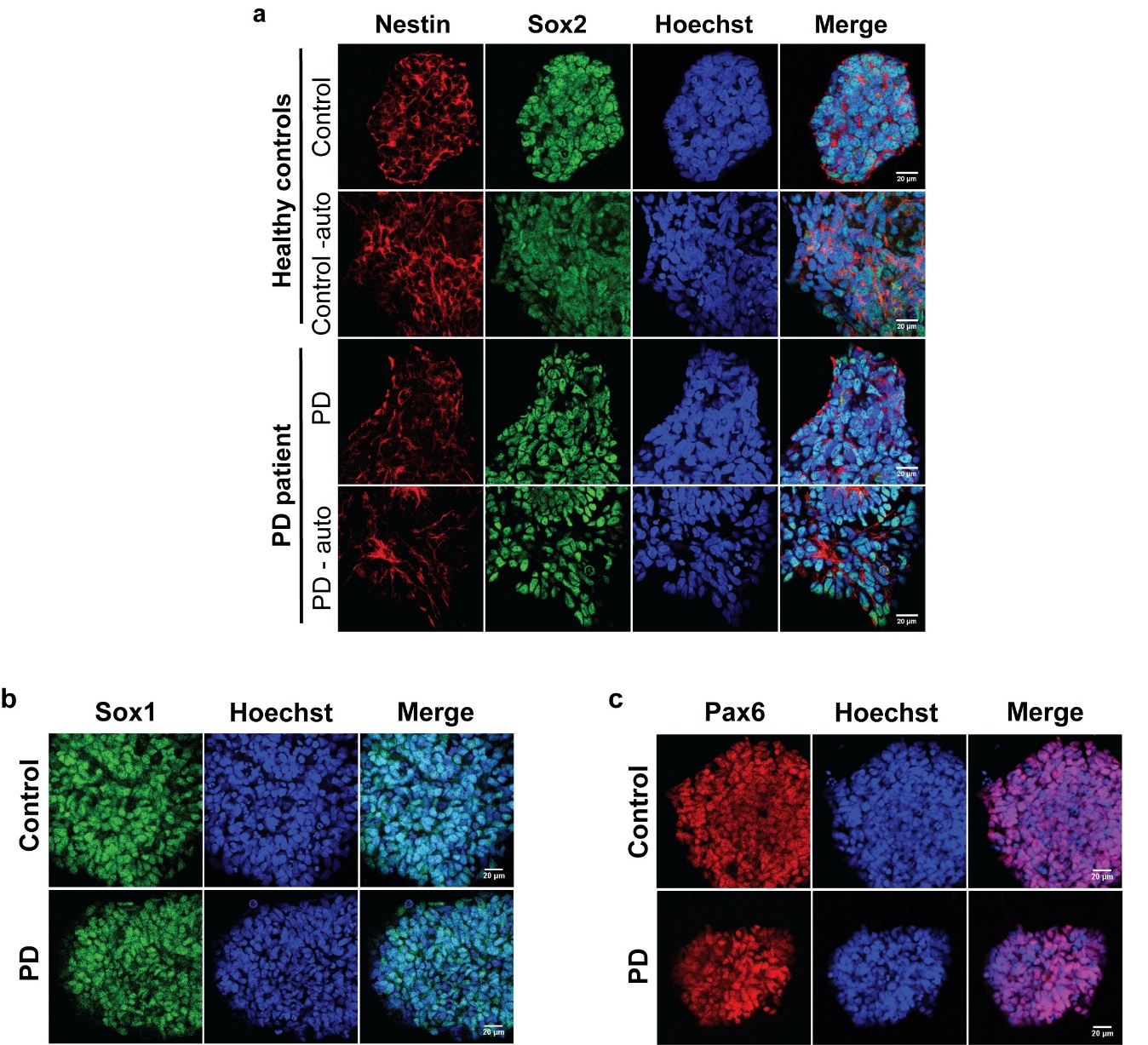


**Supplementary Figure 1 - Neuroepithelial stem cells (NESCs) characterization.** a) Representative immunostainings of Control, Control – auto, PD and PD – auto NESCs, showing expression of Nestin and Sox2. b,c) Representative immunostainings of Control and PD NESCs, showing expression of b) Sox1 and c) Pax6. Scale bar: 20 µm.

***Supplementary Figure 2***


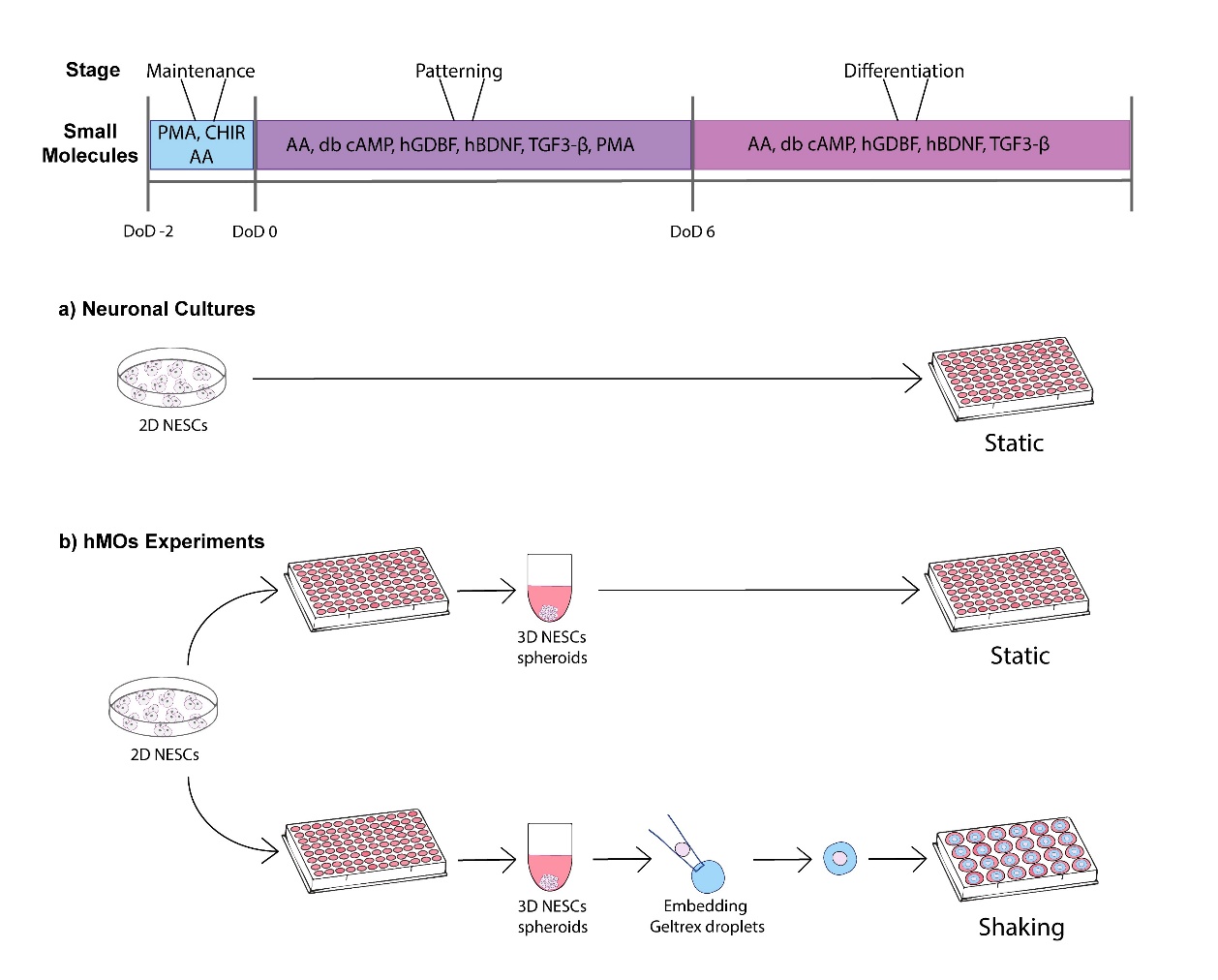


**Supplementary Figure 2 - Schematic overview of the protocol used for the generation of neuronal cultures and hMOs.**

***Supplementary Figure 3***


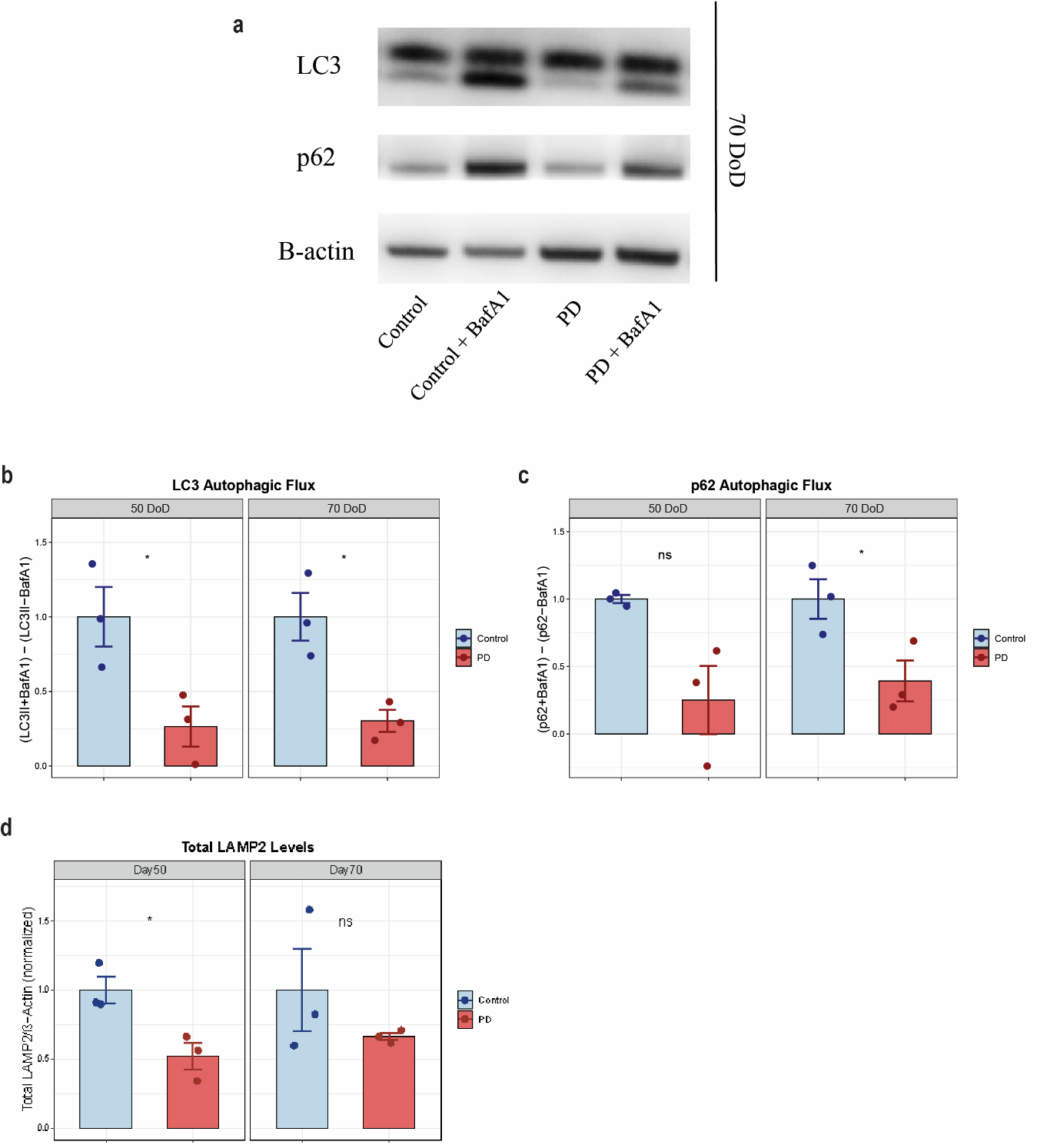


**Supplementary Figure 3 - PD hMOs showed autophagic flux dysfunction.**

(a) Representative Western blot images showing LC3, p62, and β-Actin in control and PD hMOs, treated with Bafilomycin A1 (BafA1), an inhibitor of autophagosome-lysosome fusion, for 24 h.

(b-d) Measurements of (b) LC3 flux and (c) p62 flux in hMOs after the treatment with BafA1. PD hMOs showed decreased autophagic flux compared to controls. (d) Quantification of total LAMP2 levels by Western blot. Data represent results from three independent experiments. Values are normalized to the average of controls at each time point. Statistical analysis was performed using Wilcoxon T-test; *p < 0.05, with "ns" indicating non-significant results.

***Supplementary Figure 4***


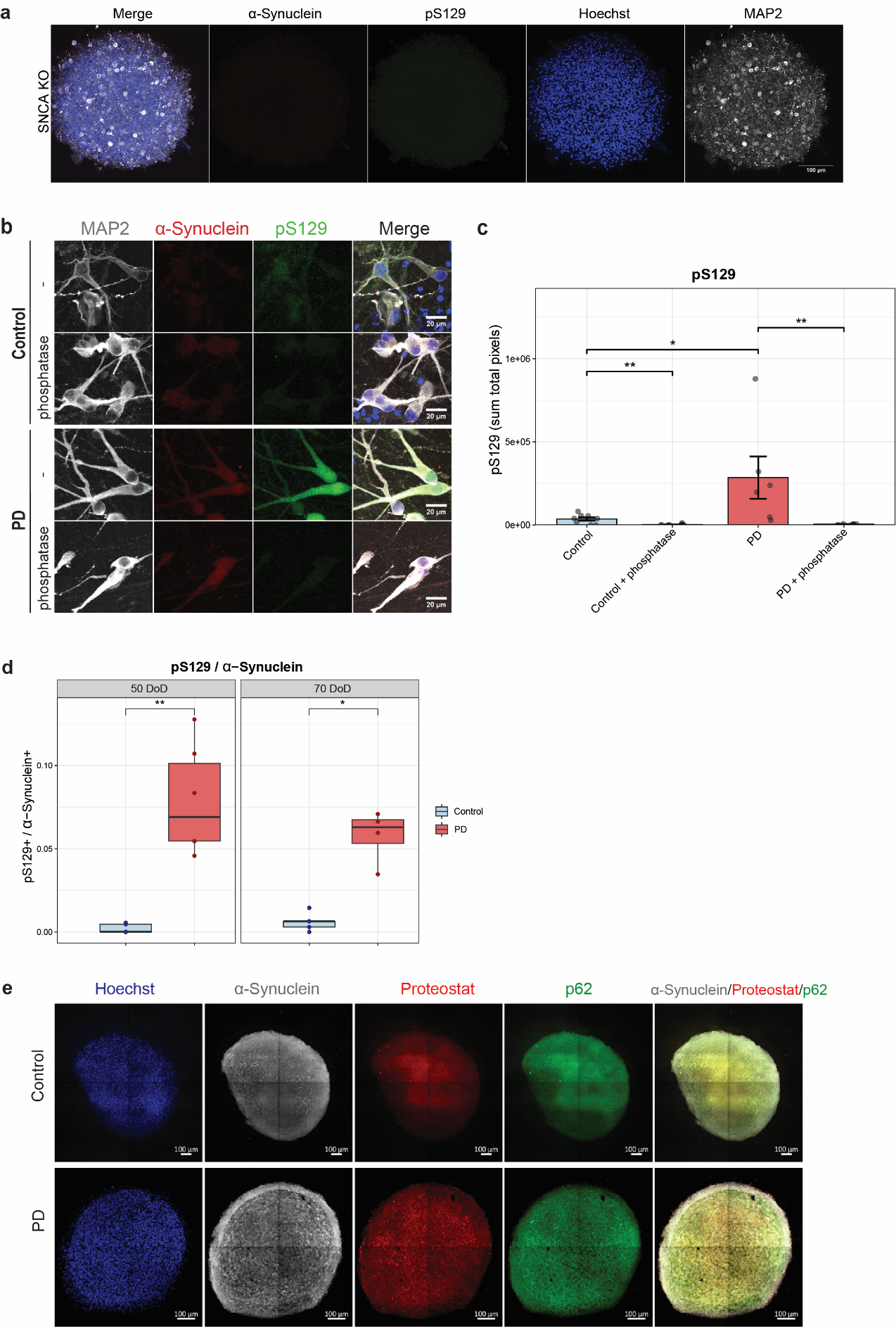


**Supplementary Figure 4 - pS129 pathology and antibody validation in PD hMOs.**

(a) The specificity of the stainings was confirmed by the absence of signal for pS129 or α-Synuclein in the SNCA KO sections. Scale bar: 100 μm.

(b, c) (b) Representative confocal images (60x) of MAP2 (grey), total α‑Synuclein (red) and pS129 (green) immunostaining in control and PD hMOs derived from midbrain floor plate neural progenitor cells (mfNPCs), either untreated or treated with Lambda phosphatase, at 70 DoD. Lambda phosphatase treatment abolishes pS129 immunoreactivity. Scale bar: 20 μm. (c) Quantification of intracellular pS129 fluorescence intensity in control and PD hMOs at 70 DoD, with and without Lambda phosphatase treatment. Data represent results from three independent experiments. Values are normalized to the average of the control. Error bars represent SEM. Image analysis was performed using a custom MATLAB script for automated quantification. Statistical analysis was performed using Mann-Whitney U test; *p < 0.05, **p < 0.01.

(d) Ratio of pS129 / total α-Synuclein in PD and control hMOs at 50 and 70 days of differentiation (DoD), analysed by immunostaining. Data result from three independent experiments. Values are normalized to the average of controls. Statistical analysis was performed using Wilcoxon T-test; *p < 0.05, **p < 0.01. Image analysis was performed using a custom MATLAB script.

(e) Representative immunofluorescence images of 70 DoD hMOs stained for nuclei (blue), α-Synuclein (grey), Proteostat (red) and p62 (green). Scale bar: 100 µm.

***Supplementary Figure 5***


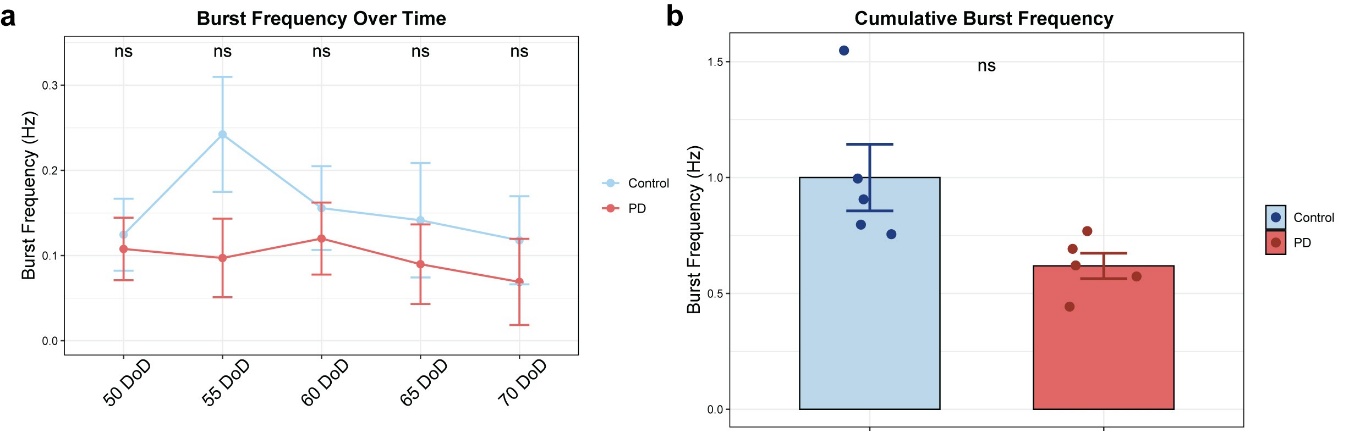


**Supplementary Figure 5 - PD hMOs exhibit a non-significant trend toward reduced burst dynamics.** a) Burst frequency in PD and control hMOs recorded every 5 days between 50 and 70 DoD. b) Cumulative burst frequency over the same period, suggesting lower overall neuronal activity in PD hMOs. Data represent the average of five independent experiments. Values were normalized to the mean of controls at each time point. Statistical analysis was performed using the Wilcoxon test, with "ns" indicating non-significant results. Error bars represent SEM. Burst detection and analysis were performed using automated MATLAB-based tools.

***Supplementary Figure 6***


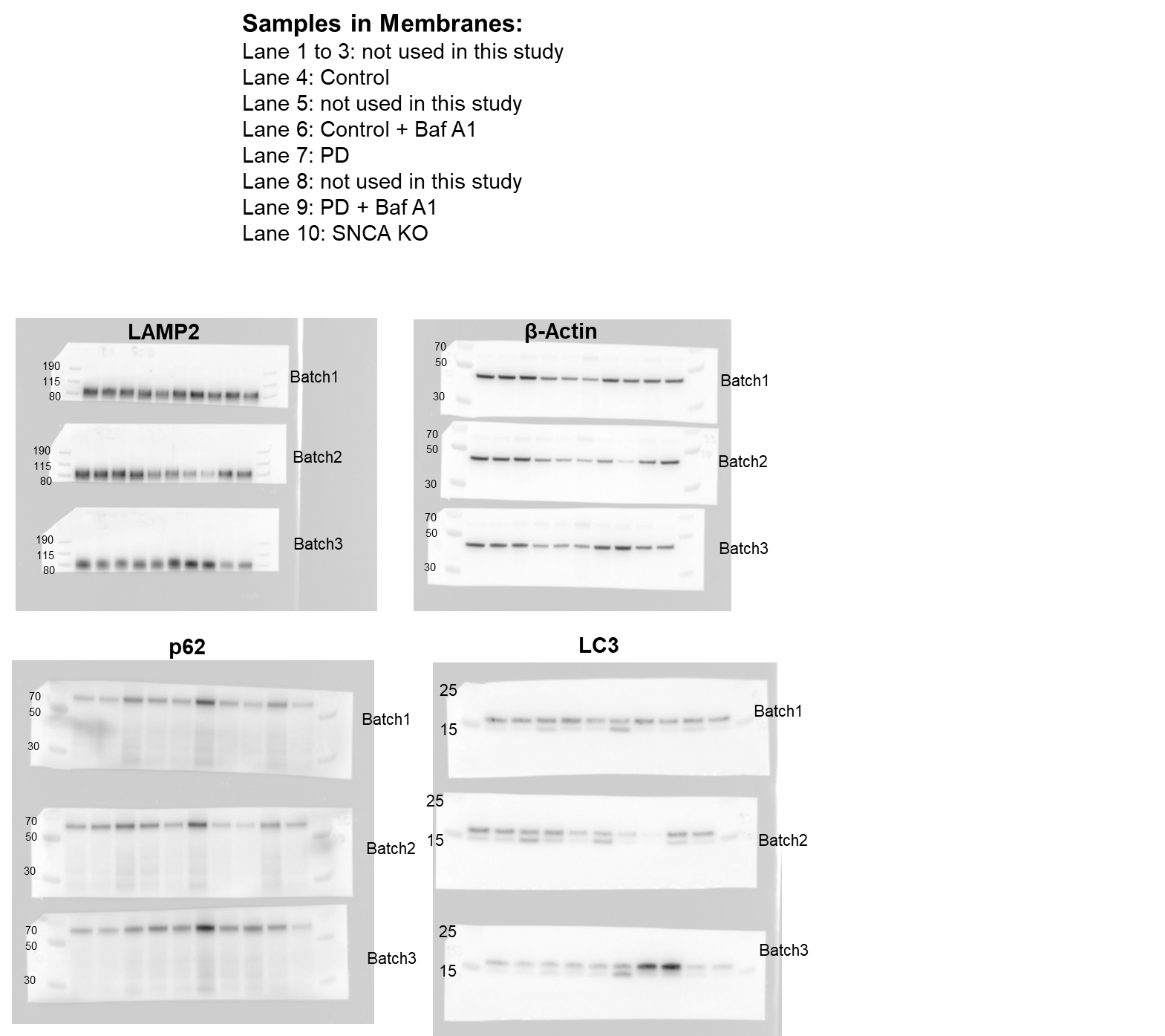


**Supplementary Figure 6 - Unprocessed original Western blot scans used in Supplementary Figure 3.** Unedited scans of all Western blot membranes used for the autophagy analysis at 50 DoD. Lane assignments and sample identification are detailed above. Molecular weight markers are shown on each membrane. “Batch1”, “Batch2”, and “Batch3” refer to independent biological replicates on separate membranes/gels, where Batch1 = Membrane 1, Batch2 = Membrane 2, Batch3 = Membrane 3. Each target protein (LAMP2, β-Actin, p62, LC3) was then incubated on the indicated membranes which were horizontally cut.

***Supplementary Figure 7***


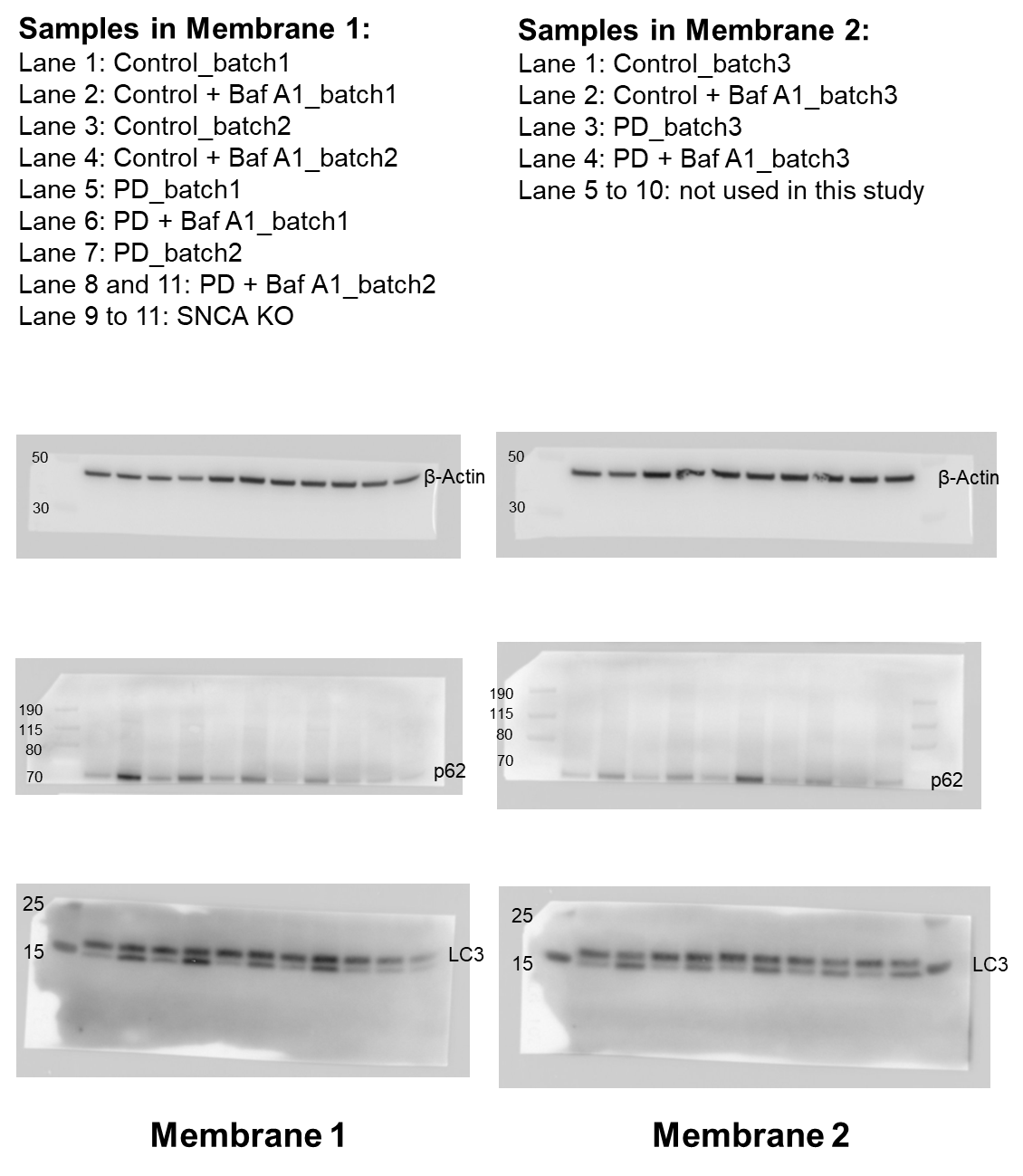


**Supplementary Figure 7 - Unprocessed original Western blot scans used in Supplementary Figure 3.** Unedited scans of Western blot membranes used for the autophagy analysis at 70 DoD. Lane assignments and sample identification are detailed above. Molecular weight markers are shown on each membrane. Each target protein (β-Actin, p62, LC3) was incubated on the indicated membranes which were horizontally cut.

***Supplementary Figure 8***

***
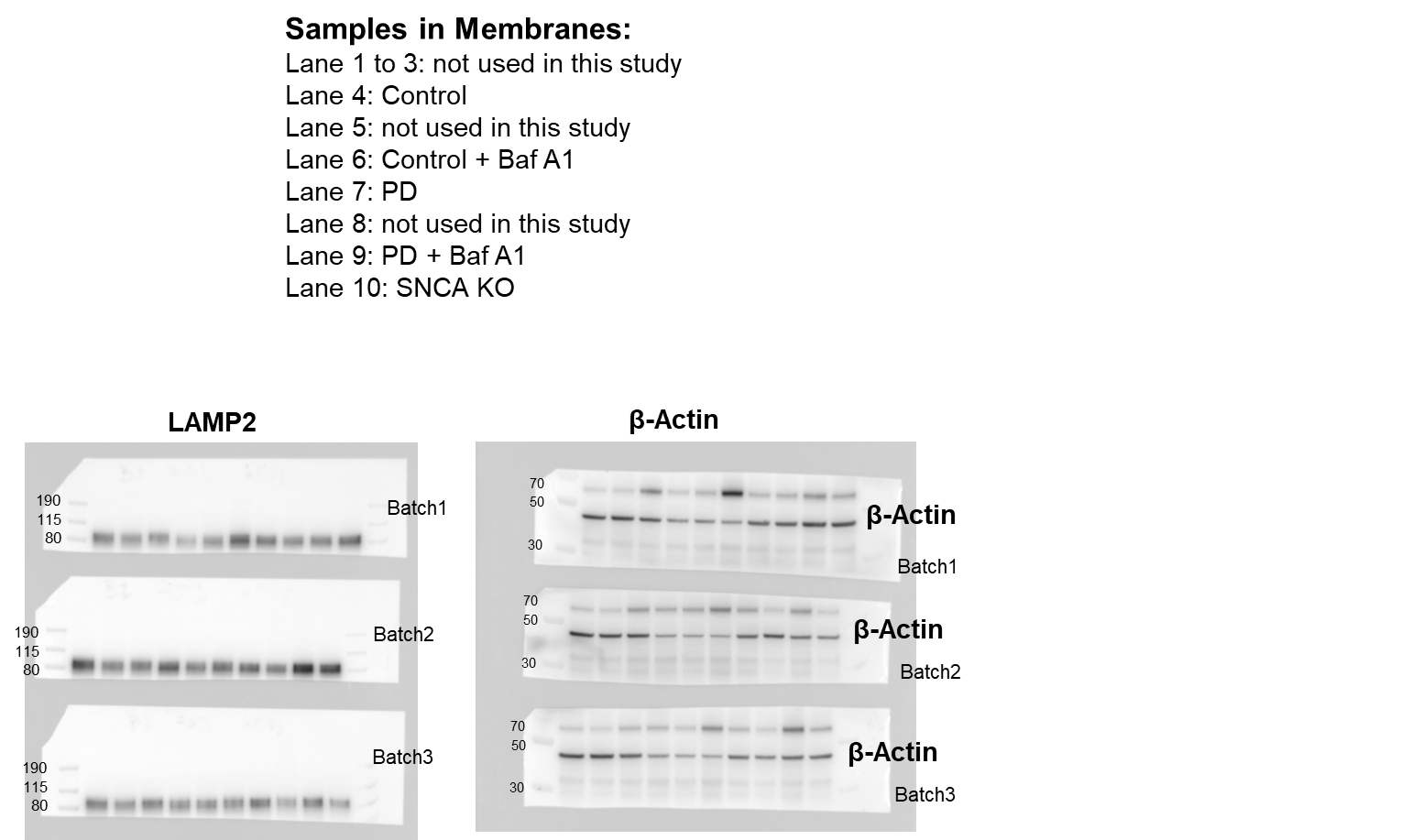
***

**Supplementary Figure 8 - Unprocessed original Western blot scans used in Supplementary Figure 3.** Raw, unedited scans of Western blot membranes used for the quantification of LAMP2 levels at 70 DoD. Lane assignments and sample identification are detailed above. Molecular weight markers are shown on each membrane. “Batch1”, “Batch2”, and “Batch3” refer to independent biological replicates on separate membranes/gels, where Batch1 = Membrane 1, Batch2 = Membrane 2, Batch3 = Membrane 3. Each target protein (LAMP2, β-Actin) was then incubated on the indicated membranes which were horizontally cut.

***Supplementary Figure 9***


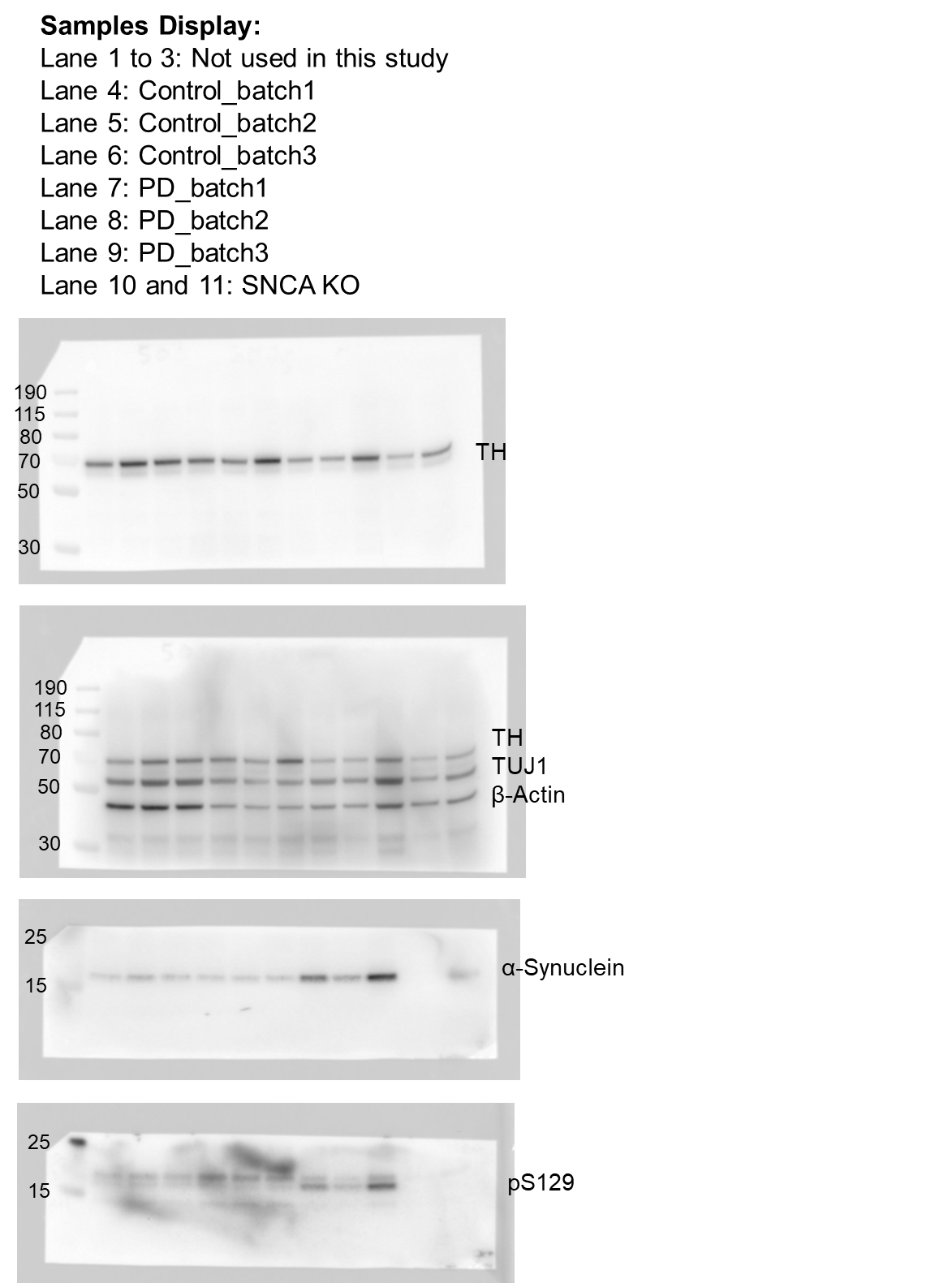


**Supplementary Figure 9 - Unprocessed original Western blot scans used in Figures 3 and 5.** Unedited scans of Western blot membranes from 50 DoD hMOs. Lane assignments and sample identification are detailed above. Molecular weight markers are shown on each membrane. Each target protein (β-Actin, TH, TUJ1, α-Synuclein, pS129) was then incubated on the indicated membranes which were horizontally cut. The 2^nd^ picture shows the same cut membrane as the 1^st^ picture but after incubation of the target protein TUJ1 and β-Actin. pS129 signal was imaged after membrane stripping and re-incubation.

***Supplementary Figure 10***

***
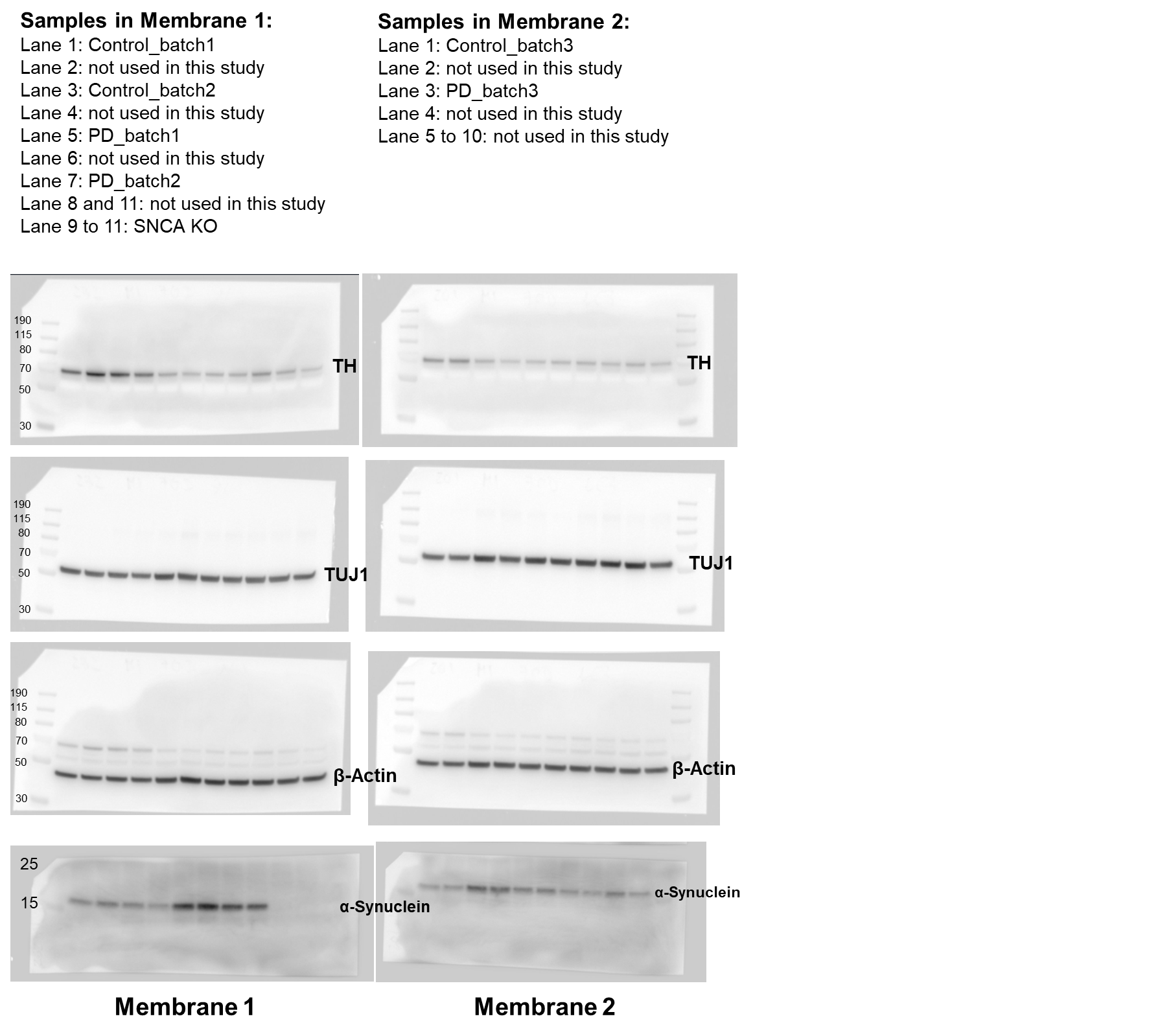
***

**Supplementary Figure 10 - Unprocessed original Western blot scans used in Figures 3 and 5.** Unedited scans of Western blot membranes from 70 DoD hMOs. Lane assignments and sample identification are detailed above. Molecular weight markers are shown on each membrane. Each target protein (β-Actin, TH, TUJ1, α-Synuclein) was then incubated on the indicated membranes which were horizontally cut. The 2 membranes were first incubated TUJ1 and then stripped to be probed by TH followed by β-Actin.

***Supplementary Figure 11***


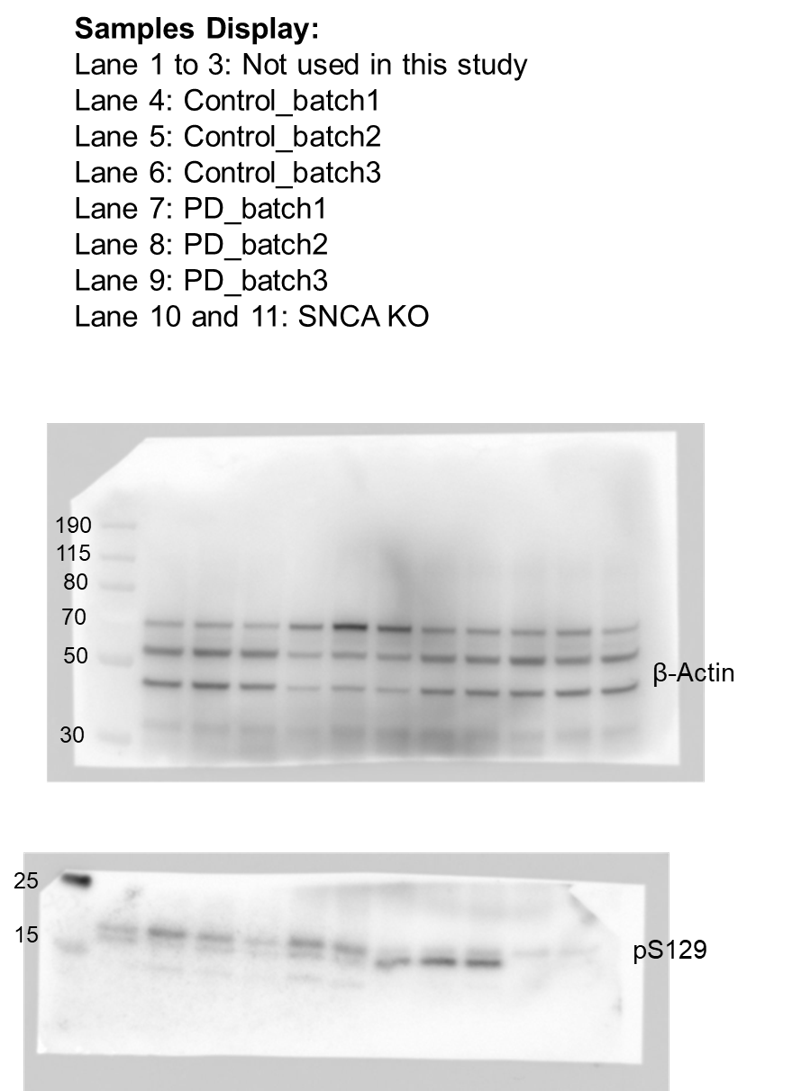


**Supplementary Figure 11 *-* Unprocessed original Western blot scans used in Figure 3.** Raw, unedited scans of Western blot membranes used for the quantification of pS129 levels at 70 DoD. Lane assignments and sample identification are detailed above. Molecular weight markers are shown on each membrane. Each target protein (pS129, β-Actin) was then probed on the indicated membranes which were horizontally cut.

Supplementary Table 1. Human iPSCs used in this study

| **Cell Line Identifier** | **Autophagy sensor LC3-Rosella** | **Cell Line Name** | **Healthy (WT) or Patient (PD) Origin** | **Sex** | **Age of sampling** | **Age of onset** | **Source** | **Ref #** | **Karyotype** |
| --- | --- | --- | --- | --- | --- | --- | --- | --- | --- |
| DB 232 | No | Control | WT | F | 55 | - | Reinhardt et al. 2013 |  | Normal |
| DB 336 | No | PD | PD | F | 55 | 50 | EBISC - EDi001-A | EDi001-A | Normal |
| DB 374 | No | SNCA KO | PD | F | 55 | 50 | Chen et al. 2019 | AST23-4KO-5B | Normal |
| DB 284 | Yes | Control - auto | WT | F | Cord  Blood  derived | - | GIBCO | A13777 | Normal |
| DB 355 | Yes | PD - auto | PD | F | 55 | 50 | EBISC - EDi001-A | EDi001-A | Normal |

Supplementary Table 2. Primary antibodies used in this study

| **Antibodies** | **Species** | **Source** | **Reference** | **RRID** | **WB Dilution** | **IF Dilution** |
| --- | --- | --- | --- | --- | --- | --- |
| α-Synuclein | Rabbit | Santa Cruz | sc7011-R | AB_2192953 | 1:1000 | 1:1000 |
| α-Synuclein (2A7) | Mouse | NOVUS  Biologicals | NBP1-05194 | AB_1555287 | - | 1:1000 |
| β-Actin | Mouse | Cell Signaling | 3700 | AB_2242334 | 1:50.000 | - |
| LAMP2 | Rabbit | Proteintech | 27823-1-AP | AB_2880983 | 1:2000 | - |
| LC3 | Rabbit | MBL | PM036 | AB_2274121 | 1:1000 | - |
| MAP2 | Chicken | Abcam | ab92434 | AB_2138147 | - | 1:1000 |
| Nestin | Mouse | Millipore | MAB5326 | AB_2251134 | - | 1:100 |
| Pax6 | Rabbit | Covance | PRB-278P | AB_291612 | - | 1:300 |
| Phospho-α-Synuclein  (Ser129)  (D1R1R) | Rabbit | Cell Signaling | 23706S | AB_2798868 | 1:500 | 1:500 |
| p62  (SQSTM1) | Mouse | Abcam | ab56416 | AB_945626 | 1:500 | 1:500 |
| Sox1 | Goat | R&D Systems | AF3369 | AB_2239879 | - | 1:200 |
| Sox2 | Goat | R&D Systems | AF2018 | AB_355110 | - | 1:200 |
| TH | Rabbit | Abcam | ab112 | AB_297840 | 1:1000 | 1:1000 |
| TUJ1 | Mouse | BioLegend | 801201 | AB_2313773 | 1: 50.000 |  |

Supplementary Table 3. Additional iPSC lines used exclusively for λ‑phosphatase antibody‑validation experiment

| **Cell Line Identifier** | **Cell Line Name** | **Healthy (WT) or Patient (PD) Origin** | **Sex** | **Age of sampling** | **Source** | **Karyotype** |
| --- | --- | --- | --- | --- | --- | --- |
| DB 200 | Control | WT | F | 81 | Reinhardt et al. 2013 | Normal |
| DB 302 | Control | WT | F | 68 | Universitats klinikum Tuebingen | Normal |
| DB 317* | PD | PD | F | 55 | Coriell ND27760 | Abnormal |

* Same donor as DB 336 (Supplementary Table 1), independent clone
